# Statistical Relational Learning With Unconventional String Models

**DOI:** 10.3389/frobt.2018.00076

**Published:** 2018-07-03

**Authors:** Mai H. Vu, Ashkan Zehfroosh, Kristina Strother-Garcia, Michael Sebok, Jeffrey Heinz, Herbert G. Tanner

**Affiliations:** ^1^Department of Linguistics and Cognitive Science, University of Delaware, Newark, DE, United States; ^2^Cooperative Robotics Lab, Department of Mechanical Engineering, University of Delaware, Newark, DE, United States; ^3^Department of Linguistics and Institute of Advanced Computational Science, Stony Brook University, Stony Brook, NY, United States

**Keywords:** statistical relational learning, Markov logic networks, grammatical inference, formal language theory, model theory, phonology, robotics, control and planning

## Abstract

This paper shows how methods from statistical relational learning can be used to address problems in grammatical inference using model-theoretic representations of strings. These model-theoretic representations are the basis of representing formal languages logically. Conventional representations include a binary relation for order and unary relations describing mutually exclusive properties of each position in the string. This paper presents experiments on the learning of formal languages, and their stochastic counterparts, with unconventional models, which relax the mutual exclusivity condition. Unconventional models are motivated by domain-specific knowledge. Comparison of conventional and unconventional word models shows that in the domains of phonology and robotic planning and control, Markov Logic Networks With unconventional models achieve better performance and less runtime with smaller networks than Markov Logic Networks With conventional models.

## 1. Introduction

This article shows that statistical relational learning (Getoor and Taskar, 2007; Domingos and Lowd, 2009; Natarajan et al., 2015) provides a natural solution to the problem of inferring formal languages when the alphabetic symbols underlying the formal languages share properties.

Formal languages are sets of strings or probability distributions over strings (Hopcroft and Ullman, 1979; Kracht, 2003; Kornai, 2007). We use the word *word* synonymously with *string*. They have found application in many domains, including natural language processing, robotic planning and control (Fu et al., 2015), and human-robot interaction (Zehfroosh et al., 2017). In each of these domains there are instances where formal languages have to be inferred from observations. Grammatical inference algorithms (de la Higuera, 2010; Heinz and Sempere, 2016) address the problem of learning formal languages in theory and practice and have found success in the aforementioned domains (Fu et al., 2015; Heinz et al., 2015).

However, there is an important, unexamined assumption in much of the grammatical inference literature. Formal languages depend on an *alphabet* of symbols, from which the strings are built. Broadly speaking, these alphabetic symbols are treated as uniformly independent. But in many domains these symbols represent entities which may share properties, and these shared properties may ease the inference problem. In other words, it may not always be appropriate to represent strings as a sequence of independent symbols. The appropriate representation of strings in a given domain may carry richer information that is kept out of view with conventional representations of strings.

In this article, we apply finite model theory (Hodges, 1993; Libkin, 2004) to study different representations of strings and show how statistical relational learning can be used to infer formal languages using these representations. Since formal languages can be expressed with logical expressions (Büchi, 1960; Thomas, 1997), it makes sense to make the logical expressions the targets of learning. This avenue has not been extensively pursued within the grammatical inference tradition, which tends to focus on representing formal languages with automata and formal grammars (de la Higuera, 2010; Heinz and Sempere, 2016). With few exceptions, both automata and formal grammars treat symbols autonomously. However, if one *were* to develop learning algorithms for logical expressions within the grammatical inference tradition, we expect the result would be precisely the kind of work present in the tradition of statistical relational learning! It is in this way that this work reveals connections between statistical relational learning, model theory, and grammatical inference.

Specifically, this article re-examines the unary relations that make up word models. These are typically assumed to be disjoint: in a string with three positions like *abc*, a position *x* cannot satisfy both a(*x*) and b(*x*). In other words, *x* cannot simultaneously be labeled both *a* and *b*.

However, in natural languages (and often in robot planning), events in a sequence can share certain properties. For instance, in the word *impossible*, it is significant that the *m* and the *p* both involve lip movement in addition to a full stoppage of the airflow in the oral cavity (Odden, 2014). Hence position *x* corresponding to either *m* or *p* could be said to satisfy the predicates labial(*x*) and stop(*x*). However, production of *m* makes air flow through the nasal cavity, unlike with *p*. So positions *x* corresponding to *m* would satisfy nasal(*x*) but positions *x* corresponding to *p* would not. In this scenario, a position *x* which simultaneously satisfies predicates labial, nasal, and stop would be interpreted as the speech sound which we express with the single symbol *m*. Similarly, an aerial robot can execute the same controlled action under different conditions, e.g., it can fly in free space, as well as in proximity to ceiling, ground, or wall; yet the aerodynamics in each case can be significantly different (Karydis et al., 2015). A ground robot that can autonomously navigate can still do so while pushing an object (Parker, 1994) or carrying a load (Kiener and von Stryk, 2007)—cf. (Mellinger et al., 2013); in each case, the dynamics of the vehicle and the effect of its action on the environment are different. Thus a robot's mode of operation is similarly characterized by a particular combination of attributes and features.

There is already precedent for the importance of the representations of words for understanding the complexity of subregular formal languages (Thomas, 1997; Rogers and Pullum, 2011; Rogers et al., 2013). For example, if the relational structures underlying word models use the successor relation (+1) to represent sequential order, then long-distance dependencies require Monadic Second Order (mso) logic to be expressed, unlike local dependencies which only require First-Order (fo) logic. Consequently, formal languages expressing local dependencies are more efficiently expressed and learned compared to those expressing long-distance dependencies *with the successor representation*. Conversely, if relational models underlying strings use the precedence relation (<) to represent sequential order, then certain kinds of long-distance dependencies can be expressed with Propositional (pr) logic, while those involving local dependencies require fo logic. Again it follows that formal languages expressing long-distance dependencies are more efficiently expressed and learned compared to those expressing local ones *with the precedence representation*. These facts are reviewed in more detail in section 4, and lends support for the idea familiar to modern artificial intelligence (ai) research, that in learning, representations *matter*.

We thus take advantage of domain-specific knowledge to model strings with carefully chosen sets of unary relations that capture salient properties. We show that doing so concretely simplifies the formal languages that are often learning targets, and makes it possible to reliably infer them with *less* data. We demonstrate this approach by applying Markov Logic Networks (Richardson and Domingues, 2006; Domingos and Lowd, 2009) to case studies drawn from the phonology of natural languages and robotic planning.

This article is organized as follows. Section 2 reviews model theory and fo logic and section 3 (MLNs). Section 4 reviews foundational aspects of formal language learning from both a categorical and probabilistic perspective. This section also introduces conventional word models and presents examples which illustrate how the character of the logical expression for a given formal language changes as a result of the model by reviewing well-studied subregular classes. Section 5 explains how well-motivated unconventional word models fit into the picture developed so far.

The remainder of the article details our experiments and contributions with Markov logic network (mln)s. Section 6 explains general features of how we employed the software package Alchemy to learn stochastic formal languages.

Section 7 presents the first experimental contribution of this paper: an empirical demonstration on a toy problem that a mln can emulate a smoothed n-gram model (Jurafsky and Martin, 2008) using a conventional string representation. A theoretical result in the form of a mathematical proof establishing the equivalence of n-gram models with these mlns is left for future research. We then postulate that if statistical relational learning modules can effectively learn formal languages expressed with conventional word models as was the case with the toy problem here, then they should also succeed for unconventional word models because the conventional word model is just one of many possible representations of strings. Thus, the results in this section give us confidence that applying mlns with unconventional word models to the problem of learning formal languages would also be meaningful and successful.

Our second contribution comes from the domain of phonology. We examine *unbounded stress assignment*, which is a long-distance dependency in well-formed words in some languages (Hayes, 1995; van der Hulst et al., 2010). As explained in Section 8, stress in phonology refers to the syllables which are pronounced prominently. We train mlns based on both conventional and unconventional word models. The unconventional word model takes into account phonological representations of stress, unlike the conventional model. Our analysis shows that the mlns with the unconventional word model generalizes more successfully on small datasets than mlns with the conventional word model.

The third contribution is found in Section 9, where statistical relational learning is applied for the first time on a problem of (deliberate) cooperative interaction between heterogeneous robots. The first objective here is first to demonstrate how the same theory that helps us reason about words and stress, can also apply to engineering problems of planning and decision making in robotics; the second objective is to show how the use of unconventional models can both analytically and computationally facilitate the analysis of cooperative interaction between autonomous agents. The case study featured in Section 9 involves an aerial vehicle, working together and physically interacting with a ground wheeled robot, for the purpose of allowing the latter to overcome obstacles that it cannot by itself. The focus in this case study is not on learning different ways in which the vehicles can interact with each other — not on the planning of the interaction per se; this can be a subject of a follow-up study.

Instances of problems where physical interaction between autonomous agents has to be coordinated and planned to serve certain overarching goals, are also found in the context of (adaptive) robotic-assisted motor rehabilitation, which to a great extent motivates the present study. In this context, humans and robotic devices may interact both physically and socially, in ways that present significant challenges for machine learning when the latter is employed to make the robots customize their behavior to different human subjects and different, or evolving, capability levels for the same subject. One of the most important challenges faced there is that one does not have the luxury of vast amounts of training data. The algorithms need to learn reliably and fast from *small* data, and the overall goal of this paper is to highlight that the type of formal representation that is used for the world and available knowledge, does matter.

The last section 10 concludes.

## 2. Model theory and first-order logic

Model theory studies objects in terms of mathematical logic (Enderton, 2001). A model of an object is a *structure* containing information about the object. The type of information present in a model theory of a set of objects is given by the *model signature*, and a set of mathematical statements about how structures are *interpreted*. Specifically, a model signature contains a domain (a set of elements), a set of relations, and a set of functions.^1^ Here, we only consider model signatures with finite domains and whose signatures contain only relations and no functions. In other words, we apply *finite* model theory to *relational structures* (Libkin, 2004).

Model signatures define a collection of *structures* S (or models, or representations), which are tuples consisting of a finite domain *D*, and a finite number *m* ∈ ℕ of *n*_*i*_-ary relations *R*_*i*_, for 1 ≤ *i* ≤ *m* and *n*_*i*_ ∈ ℕ. A structure is therefore denoted $S=\langle D;R_{1},R_{2},\ldots ,R_{m}\rangle$. For a finite domain *D*, its elements are standardly given as elements of ℕ: *D* = {1, …*k*} for some *k* ∈ ℕ. The *size* of S, denoted |S|, coincides with the cardinality of its domain. In the context of this paper, the model signature, denoted 𝔐 = 〈𝔇; ℜ〉, specifies what kind of elements and relations are present in a structure. Here, 𝔇 is a set of domains, and ℜ is a set of relations in a particular structure. Thus a structure S of signature 〈𝔇; ℜ〉 will have *D* ∈ 𝔇 and 〈*R*_1_, …, *R*_*m*_〉 ∈ ℜ, in other words, structures are specific instantiations of some particular signature. Such instantiations are referred to as *groundings*. Given a finite set *C* of constants (domain elements), the Herbrand base of all the possible groundings of the relations in ℜ with respect to *C* is *H*_*C*_ = {*R*(*c*_1_…*c*_*n*_)∣*R*is an *n*-ary relation inℜ, *c*_*i*_ ∈ *C*}.

A signature gives rise to a fo logical language where the names of the relations in ℜ become atomic predicates in the logic. In fo logic, there are variables *x, y, z*… which range over the elements in the domain of the structure. The logical language has a syntax to define sentences. These are usually defined inductively with the predicates and variable equality (=) serving as base cases, and with the inductive cases provided by Boolean connectives (∧, ∨, →, ↔) between formulas, in addition to quantification (∃, ∀) over formulas. The logical language also has a semantics, which lets one determine whether a well-formed logical formula φ is true for some structure S. This semantics is compositional and can be computed following the syntactic structure of φ. We assume some previous familiarity with fo logic. Enderton (2001) and Libkin (2004) provide good references for formal treatments.

## 3. Markov logic networks

We adapt the presentation of De Raedt et al. (2016). A *Markov Network* is a representation of a Markov random field (Pearl, 1988). It expresses graphically the joint distribution of a collection of random variables *X* = {*X*_1_, *X*_2_, …*X*_*n*_} taking values in some space X. Here, these random variables are assumed discrete and finite. A Markov Network representation consists of an undirected graph and a set of potential functions ϕ_*k*_. There is one such potential function ϕ_*k*_ for every clique in the graph, and the clique associated with potential function ϕ_*k*_ is denoted {*k*}. The subset of random variables associated with that clique is denoted *X*_{*k*}_.

If a particular valuation of *X* is denoted *x*, and given that X is finite, one can define the *partition function*

(1) $$\begin{array}{l} Z=\underset{x\in X}{\sum }\underset{k}{\prod }\phi_{k}(x_{\left\{k\right\}}) \end{array}$$

Then the joint probability distribution of the network can be factored over the network's cliques in the form

$$\begin{array}{l} \text{P}\left(X=x\right)=\frac{1}{Z}\underset{k}{\prod }\phi_{k}(x_{\left\{k\right\}}) \end{array}$$

Usually, a log-normal representation for this joint probability distribution is utilized, in the form of an exponential of a weighted sum of real-valued feature functions *f*_*j*_(*x*). There is one such feature *f*_*j*_ for each possible valuation of the state *x* in clique *k*, and this feature is weighted with *w*_*j*_ = logϕ_*k*_(*x*_{*k*}_). In this form, the joint probability distribution is

$$\begin{array}{l} \text{P}\left(X=x\right)=\frac{1}{Z}\text{exp}\left(\underset{j}{\sum }w_{j}f_{j}\left(x\right)\right) \end{array}$$

A mln is a set of pairs (*F*_*i*_, *w*_*i*_), where *F*_*i*_ is a first order formula and *w*_*i*_ is a real number. Note that an fo logic associated to some signature 〈𝔇; ℜ〉, naturally provides such formulas. The mln now becomes a template for generating Markov networks: given a domain *D* ∈ 𝔇 and a collection of atomic predicates 〈*R*_1_, …, *R*_*m*_〉 ∈ ℜ with signature 〈𝔇; ℜ〉, there is a node for every possible grounding of an atomic predicate *R*_*i*_, and a feature *f*_*j*_ for each possible grounding of a formula *F*_*i*_. In fact, despite being different depending on the choice of *D* and *R*_*i*_, all of these *ground* Markov networks have the same potential for a given formula *F*_*i*_, namely $\phi_{i}\left(x_{\left\{i\right\}}\right)={\text{e}}^{w_{i}}$. The feature *f*_*j*_(*x*) is equal to the number of all true groundings of formula *F*_*j*_ in *x*. It is denoted *n*_*F*_*j*__. Thus, the joint distribution of the ground Markov network generated by the mln is expressed by

(2) $$\begin{array}{l} \text{P}\left(X=x\right)=\frac{1}{Z}\underset{i}{\prod }\phi_{i}(x_{\left\{i\right\}}{)}^{n_{F_{i}}\left(x\right)}\\ =\frac{1}{Z}\underset{i}{\prod }\text{exp}(w_{i}n_{F_{i}}\left(x\right))\\ =\frac{1}{Z}\text{exp}\underset{i}{\sum }w_{i}n_{F_{i}}\left(x\right) \end{array}$$

Since each structure S corresponds to a particular instantiation of the random vector *X* = *x* given the set of formulas and weights (*F*_*i*_, *w*_*i*_), (2) essentially expresses the probability that the mln assigns to a particular structure:

(3) $$\begin{array}{l} P\left(S\right)=\frac{1}{Z}\text{exp}\underset{i}{\sum }w_{i}n_{F_{i}}\left(S\right) \end{array}$$

From a learning perspective, natural problems include finding either the weights of given formulas or both the weights and the formulas themselves (Domingos and Lowd, 2009, Chapter 4). In this paper we only concern ourselves with the former problem and assume the specific domains provide the formulas a priori.

For any parametric model *M* with a set of parameters *P* and set of data *D*, the maximum likelihood estimate (mle) refers to the parameter values $\hat{P}$ that maximize the likelihood of the *D* according to *M*. In other words any parameter values which deviate from $\hat{P}$ will result in *M* assigning a smaller probability to *D*. mlns are parametric models where the weights are the parameters. Finding the mle is thus a natural learning problem for mlns.

In principle, the weights of the formulas of a mln that yield the mle of the data can be found by adjusting their values so as to reduce the difference between the actual counts of the groundings of the formulas in the data and the expected counts given the current weights on the formulas. This is expressed with partial derivative of the log-likelihood of the data *D* below for a given set 〈*F, w*〉 of pairs of formulas and weights (*F*_*i*_, *w*_*i*_) in the mln.

$$\begin{array}{l} \frac{\partial }{\partial w_{i}}\text{log }{\text{P}}_{\langle F,w\rangle }\left(D\right)=n_{F_{i}}\left(D\right)-{\text{E}}_{\langle F,w\rangle }[n_{F_{i}}\left(D\right)] \end{array}$$

Then standard optimization techniques, such as gradient descent, the conjugate gradient, and Newton's method, or variants thereof, can be used to find weights corresponding to the mle of the data given the mln. In practice, computing $n_{F_{i}}\left(S\right)$ is challenging. The gradient of the pseudo-log-likelihood is often calculated instead as this is much more efficient. The price paid is that any guarantees of convergence to maximum likelihood are lost. Gaussian priors to prevent overfitting are also used.

## 4. Strings and stringsets

Strings (words) are familiar: they are sequences of symbols and formal languages are sets of strings. Formal language theory studies the computational nature of stringsets (Hopcroft and Ullman, 1979). Since patterns in strings can be represented with formal grammars, the question addressed by the field of grammatical inference is how grammars, such as automata, can be learned under various learning paradigms (de la Higuera, 2010; Heinz and Sempere, 2016). However, stringsets can also be expressed with logic. Learning these logical expressions is therefore another strategy for inference. This is where relational learning, statistical relational learning, and related fields like Inductive Logic Programming become relevant.

In this section, we provide formal background and notation on strings, formal languages, finite-state automata, logic, and model theory. Connections among them are made along the way.

### 4.1. Strings

In formal language theory, the set of symbols is fixed, finite and typically denoted with Σ. The free monoid Σ^*^ is the smallest set of strings which contains the unique string of length zero λ (the identity element in the monoid) and which is closed under concatenation with the symbols from Σ. Thus, if *w* ∈ Σ^*^ and σ ∈ Σ then *wσ* ∈ Σ^*^ where *wσ* represents the string obained by concatenating σ to the end of *w*. Concatenation applies between strings as well. If *u* and *v* are strings, then *uv* represents their concatenation.

For all *u, v, w, x* ∈ Σ^*^, if *x* = *uwv* then *w* is a *substring* of *x*. If $x\in \Sigma^{*}\sigma_{1}\Sigma^{*}\sigma_{2}\Sigma^{*}\ldots \sigma_{n}\Sigma^{*}$ then *w* = σ_1_σ_2_…σ_*n*_ is a *subsequence* of *x*. A substring (subsequence) of length *k* is called a *k*-factor (*k*-subsequence). Let factor_*k*_(*w*) denote the set of substrings of *w* of length *k*. Let subseq_*k*_(*w*) denote the set of subsequences of *w* up to length *k*. The domains of these functions are extended to languages in the normal way.

We sometimes make use of left and right word boundary markers (⋊ and ⋉, respectively), but do not include those in Σ.

### 4.2. Stringsets

Formal languages are subsets of Σ^*^. For example suppose Σ = {*a, b*} and consider the set of strings which contains an even number of *a*s, which we denote *E*_*a*_. *E*_*a*_ is a subset of Σ^*^. It is useful to identify every formal language *S* ⊆ Σ^*^ with its characteristic function $f_{S}:\Sigma^{*}\to \left\{0,1\right\}$. Continuing the example, if *w* = *abaaa*, then *f*(*w*) = 1 since the string *w* has an even number of *a*s and so belongs to *E*_*a*_, but if *w* = *abbaa*, then *f*(*w*) = 0 since it has an odd number of *a*s and does not belong to *E*_*a*_. This shift in perspective provides a direct parallel to the study of probability distributions over Σ^*^. These are expressed as functions whose co-domains are the real-interval [0, 1]. Formally they are *f*:Σ^*^ → [0, 1] such that $\underset{w\in \Sigma^{*}}{\sum }f\left(w\right)=1$. In other words, sets of strings and probability distributions over strings are identified as functions with domain Σ^*^. We use the term *categorical stringsets* to refer to subsets of Σ^*^ identified with *f*:Σ^*^ → {0, 1} and the term *stochastic stringsets* to refer to subsets of Σ^*^ identified with *f*:Σ^*^ → [0, 1]. We use the *stringset* to refer to both categorical and stochastic ones.

One important problem studied addressed in formal language theory is the membership problem, which is the problem of deciding whether an arbitrary string in Σ^*^ belongs to a categorical stringset. A closely related problem is determining the probability of an arbitrary string in a stochastic stringset. In each case, the problem is, for all *w* ∈ Σ^*^, to compute the output of *f*(*w*).

These functions *f* may be learned from examples. There are different ways the learning problem can be formulated (Jain et al., 1999; De Raedt, 2008; de la Higuera, 2010; Clark and Lappin, 2011; Heinz, 2016). One way is to require that the data sample input to the learning algorithms only contains *positive evidence*. For functions *f*:Σ^*^ → {0, 1} this means the evidence only contains words *w* such that *f*(*w*) = 1. For functions *f*:Σ^*^ → [0, 1] which are probability distributions this usually means the evidence is obtained according to independent and identically distributed (i.i.d.) draws from *f*.

Both the membership and learning problems are closely related to the study of formal grammars. It is well-known, for instance, that if the functions *f* are *regular* functions then computing *f*(*w*) is straightforward.

### 4.3. Regular stringsets and automata

Informally, *regular* stringsets are those whose membership problem can be decided by a computation model whose memory is independent of the length of the input *w*. Such stringsets underlie many applications in natural language processing (Mohri, 2005) and planning and control (Kress-Gazit et al., 2009). Formally, regular stringsets can be characterized in multiple, independently motivated ways from automata theory, logic, and algebra (Thomas, 1997; Droste and Gastin, 2009).

DEFINITION 1. A real-weighted deterministic finite-state acceptor (rdfa) is a tuple (Σ, *Q, q*_0_, δ, ρ, α):

**Table:** 

Σ	is a finite alphabet of symbols,
*Q*	is a finite set of states,
*q*_0_ ∈ *Q*	is the designated start state,
δ:*Q*×Σ → *Q*	is the transition function,
ρ:*Q*×Σ → [0, 1]	is a real-valued weight, and
α:*Q* → [0, 1]	is a function mapping each state to a real-valued weight.

A (rdfa) processes strings (words) in Σ^*^ reading them from left to right, and transitioning from one state to another upon reading each of the symbols in the input string.

Each rdfa gives rise to a function *f*:Σ^*^ → [0, 1]. The function *f* associated with an rdfa *A* = (Σ, *Q, q*_0_, δ, ρ, α), and henceforth denoted *f*_*A*_, can be derived as follows. Let a dot (·) denote real number multiplication, a backslash (\) set difference. Define the function “process”

$$\begin{array}{l} \pi :Q\times \Sigma^{*}\times \left[0,1\right]\to \left[0,1\right] \end{array}$$

recursively as follows:

$$\begin{array}{l} \pi \left(q,\lambda ,r\right)=r\cdot \alpha \left(q\right)\\ \pi \left(q,wa,r\right)=\pi (\delta \left(q,a\right),w,r\cdot \rho \left(q,a\right)) \end{array}$$

In other words, π(*q, wa, r*) processes *wa* from state *q* with current value *r* by successively transitioning the rdfa *A* to the next state as given by the letter *a* and transition function δ. The value *r* is updated at each step by multiplying the real-valued weight associated with that transition ρ(*q, a*). When the process concludes at state *q*, the value *r* is multipled by α(*q*). Then *f*_*A*_ can be defined as

$$\begin{array}{l} f_{A}(w)\;\underline{\underline{\text{def}}}\;\pi (q_{0},w,1)\;. \end{array}$$

Note that *f*_*A*_(*w*) may be undefined for some *w* if δ, ρ, and α are undefined for some (*q*, σ).

The recursive path of computation given by π indicates how the membership problem for any stringset definable with a rdfa is decided. Examples are given below.

If for each state *q* ∈ *Q* it holds that

$$\begin{array}{l} \underset{\sigma \in \Sigma }{\sum }\rho \left(q,\sigma \right)+\alpha \left(q\right)=1 \end{array}$$

then *f*_*A*_ computes a probability distribution over Σ^*^ (de la Higuera, 2010). We call such an rdfa a probabilistic deterministic finite-state acceptor (pdfa) because the real-valued weights can be interpreted as probabilities. Strings *w* for which *f*_*A*_(*w*) are undefined are said to have probability 0.

**Figure 1:**
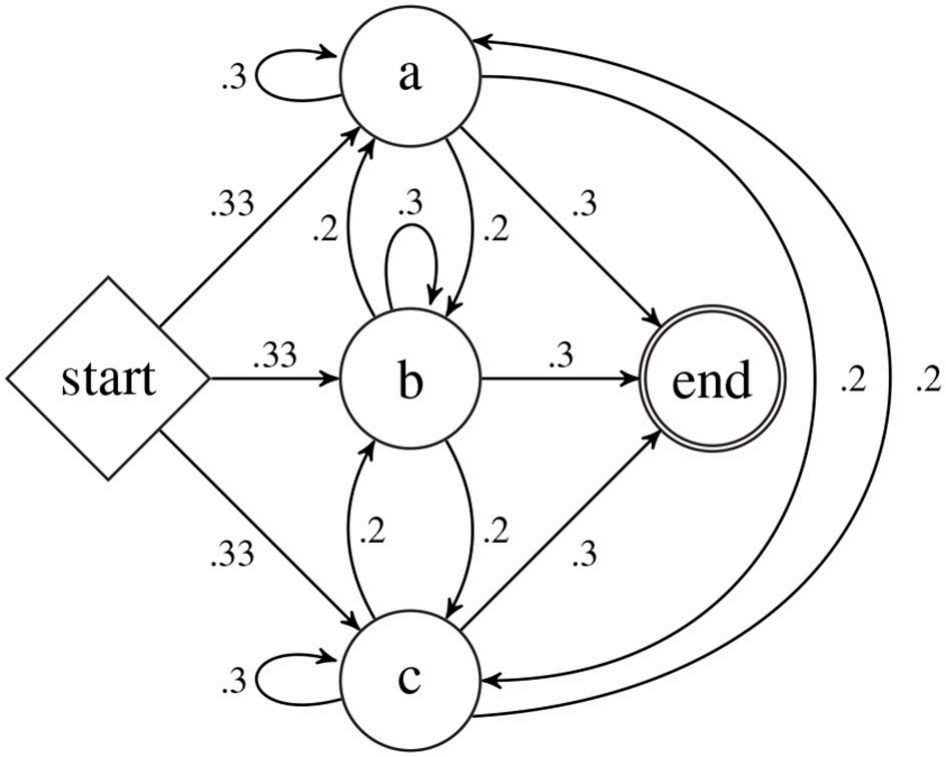
The pdfa *A* is the basis for the case study in section 7.

As an example, let Σ = {*a, b, c*} and consider the graphical representation of the pdfa *A* shown in Figure 1. This pdfa is used in the case study in Section 7. This pdfa has four states: *Q* = {a,b,c,start} indicated by the circles and diamond bearing those labels. The δ, ρ, and α functions are indicated by the arrows (which we also call *transitions*) as follows. For all *q, r* ∈ *Q*, if there is an arrow from state *q* to *r* then δ(*q, r*) = *r*. Thus from the start state, upon processing the symbol *a*, the pdfa transitions to state *a*. The numbers on the transitions between states shows the ρ function. For example, ρ(*start, a*) = 0.33 and ρ(*b, a*) = 0.2. The α function is shown with the arrows from the states to the circle labeled “end.” For example α(*a*) = 0.3. The probability *A* assigns to the string *ab* is calculated as follows.

$$\begin{array}{l} f_{a}\left(ab\right)=\pi \left(\text{start},ab,1\right)=\pi \left(a,b,1\cdot 0.33\right)=\pi \left(b,\lambda ,1\cdot 0.33\cdot 0.2\right)\\ =1\cdot 0.33\cdot 0.2\cdot 0.3=0.01980 \end{array}$$

Note the final product above correlates with the probabilities along the “path” taken by *A* when processing *ab*: 1·ρ(start, a)·ρ(a, b)·α(b).

Next we turn to rdfas for defining categorical stringsets. If for each state *q* ∈ *Q* and σ ∈ Σ it holds that ρ(*q*, σ) equals 1, 0, or is undefined and α(*q*) equals 1, 0, or is undefined, then *f*_*A*_ identifies a characteristic function of a regular categorical stringset *S* ⊆ Σ^*^. Strings *w* for which *f*_*A*_(*w*) = 1 are said to be *accepted*. Strings for which *f*_*A*_(*w*) = 0 or are undefined are said to be *rejected*; in the latter case, we let *f*_*A*_(*w*) = 0. We call such an rdfa a deterministic finite-state acceptor (dfa).

**Figure 2:**
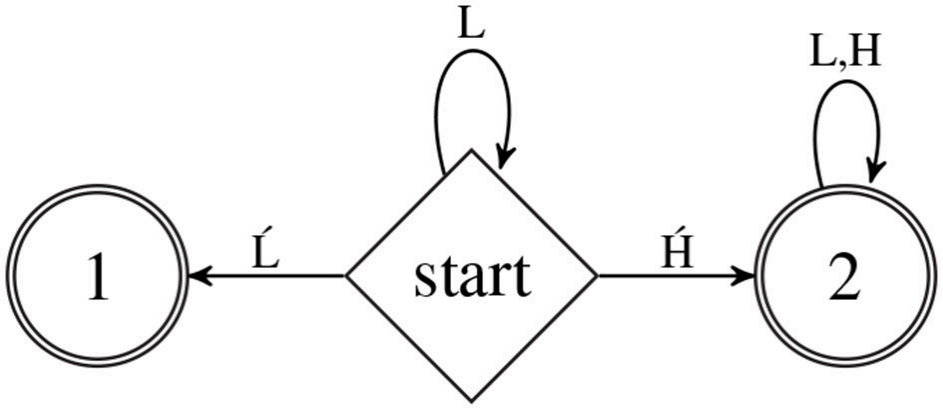
The dfa *A*_LHOR_ is the basis for the case study in section 8.

As an example, let Σ={H, $\overset{\prime }{\text{H}}$, L, Ĺ} and consider the dfa *A*_LHOR_ shown in Figure 2. The categorical stringset represented by this dfa is the basis for the case study in Section 8. In Figure 2, Q={start,1,2}, and the δ and ρ functions are as follows. For all *q, r* ∈ *Q*, if there is an arrow from state *q* to *r* labeled σ then δ(*q*, σ) = *r*. If no such arrow is present for *q*, σ then δ(*q*, σ) is undefined. Thus from the start state, upon processing the symbol $\overset{\prime }{\text{H}}$, the dfa transitions to state 2. Similarly, for all *q, r* ∈ *Q*, ρ(*q*, σ) = 1 iff there is an arrow from state *q* to *r* labeled σ; otherwise ρ(*q*, σ) = 0. The α function is defined as follows: α(1) = α(2) = 1 and α(start) = 0.

Witness the following computation of *A*_LHOR_ on input LL.

$$\begin{array}{l} f_{A_{\text{LHOR}}}\left(\text{LL}\right)=\pi \left(\text{start, LL, 1}\right)=\pi \left(\text{start, L},\;1\cdot 1\right)\\ =\pi \left(\text{start},\;\lambda ,1\cdot 1\cdot 1\right)=1\cdot 1\cdot 1\cdot 0=0 \end{array}$$

Thus *A*_LHOR_ rejects this string. On the other hand, *A*_LHOR_ accepts LĹ.

$$\begin{array}{l} f_{A_{\text{LHOR}}}\left(\text{LĹ}\right)=\pi \left(\text{start, LĹ, 1}\right)=\pi \left(\text{start, Ĺ},\;1\cdot 1\right)\\ =\pi \left(1,\lambda ,1\cdot 1\cdot 1\right)=1\cdot 1\cdot 1\cdot 1=1 \end{array}$$

The reader may verify that *A*_LHOR_ also rejects ĹH and accepts L$\overset{\prime }{\text{H}}$. The significance of what this categorical stringset represents is discussed in Section 8.

### 4.4. Learning regular stringsets

There are learning results for the general case of learning any regular stringset and results for learning subclasses of regular stringsets. An early result was that regular categorical stringsets cannot be learned exactly from positive evidence only (Gold, 1967), though they can be learned exactly from positive *and* negative evidence (Oncina and Garcia, 1992). There are also theoretical guarantees for learning regular, stochastic stringsets to any arbitrary degree of precision (Carrasco and Oncina, 1994, 1999). De la Higuera (2010) gives a comprehensive uniform presentation of such results.

Each dfa describes a class of categorical stringsets, and each stringset in this class can be learned exactly from positive evidence only (Heinz and Rogers, 2013). Similarly, each pdfa describes a class of stochastic stringsets by varying ρ and α and keeping the other aspects of the pdfa constant. One way then to express the problem of learning a stochastic stringset associated to a pdfa is to set ρ and α so that they maximize the likelihood of the data (mle). There is a simple solution to this problem which amounts to normalizing the counts of the pdfa's parsing of this data (de la Higuera, 2010).

### 4.5. Logical descriptions of stringsets

Regular stringsets can also be defined logically. Traditional logic is used for categorical stringsets and weighted logic for stochastic stringsets (Droste and Gastin, 2009). Informally, an unweighted logical expression φ picks out the strings which satisfy the condition expressed by φ. Similarly, a weighted logical expression will assign weights (for example real numbers) to strings.

In order to define a stringset with a logical expression, the logical expressions need to be able to refer to aspects and properties of the string. This is where model theory becomes relevant. Model theory makes explicit the representation of objects. Combined with a logic, such as fo or mso, a *logical language* is produced. The expressions of these logical languages define stringsets.

For example, consider the unweighted logical expression shown below, which is read as “for all *x*, it is not the case that *x* is labeled with a.”

$$\begin{array}{l} \varphi \;\underline{\underline{\text{def}}}\;\left(\forall x\right)\left[\neg \text{a}\left(x\right)\right] \end{array}$$

In plain English, this means “Well-formed words do not contain the letter a.” For example, strings like *bcb* satisfy φ since no position *x* is labeled a. However, the string *bab* does not satisfy φ because when *x* is assigned to the second position in the string, it satisfies a(*x*) and hence makes φ false.

In general, the interpretation of φ depends on what the atomic predicates are in the *models* of words. Conventional models of strings are relational structures, whose signature contains |Σ| unary relations and a single binary relation which represents the order between the elements of the string. For concreteness, let us examine two distinct conventional model-theoretic representations of words.

For the sake of this analysis let Σ = {*a, b, c*} and let the set of objects of interest be Σ^*^. Then following Rogers and Pullum (2011) and Rogers et al. (2013), one conventional model for words can be the Successor Word Model (𝔐^⊲^), which is given by the signature 〈𝔇; ⊲, *R*_*a*_, *R*_*b*_, *R*_*c*_〉 where ⊲ is the binary ordering relation *successor* and for each σ ∈ Σ, *R*_σ_ is a unary relation denoting which elements are labeled σ.

Contrast this with another conventional model for words: the Precedence Word Model (𝔐^<^). This model (structure) has signature 〈*D*; <, *R*_*a*_, *R*_*b*_, *R*_*c*_〉 where < is the binary ordering relation *precedence*, and the unary relations *R*_σ_ are the same as in 𝔐^⊲^.

Under both model signatures, each string *w* ∈ Σ^*^ of length *k* has a unique interpretable structure. The model of string *w* = σ_1_σ_2_…σ_*k*_ has domain *D* = {1, 2…*k*}, and for each σ ∈ Σ, *R*_σ_ = {*i* ∈ *D*∣*w*_*i*_ = σ}. The difference between 𝔐^⊲^ and 𝔐^<^ is the ordering relation. Under the successor model 𝔐^⊲^, the ordering relation is ⊲ $\;\underline{\underline{\text{def}}}\;$ {(*i, i* + 1) ∈ *D*×*D*}, while for the precedence model 𝔐^<^, the ordering relation is < $\;\underline{\underline{\text{def}}}\;$ {(*i, j*) ∈ *D*×*D*∣*i* < *j*}.

**Figure 3:**
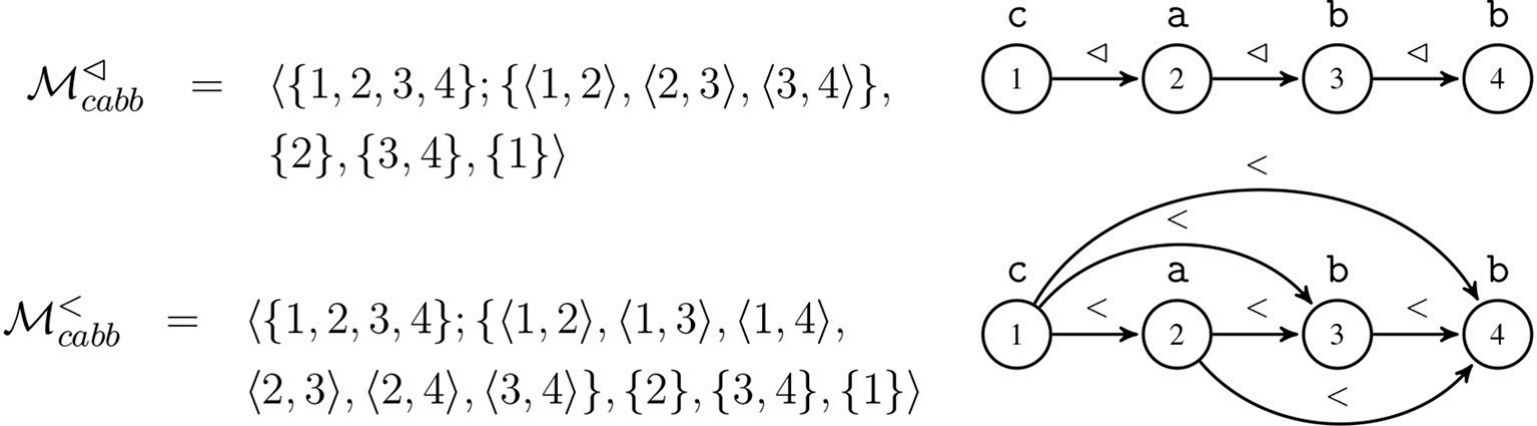
Successor and precedence models for word *cabb* with graphical representations.

Figure 3 illustrates these different word models with the word *cabb*, along with graphical representations of the models. In these graphs, nodes represent domain elements; binary relations are shown with directed labeled edges; and unary relations are shown as labels above the nodes. Note that in both models *R*_*a*_ = {2}, *R*_*b*_ = {3, 4}, and *R*_*c*_ = {1}. While the unary relations in these models illustrated in Figure 3 are the same because the same positions have the same labels, information about the order of the elements is represented differently.

It follows that certain conditions must be met for structures to be interpretable as strings. In both theories, for a structure S with domain *D* to be interpretable as a word, each element in *D* must have at least one label—symbolically translated as (∀*i* ∈ *D*)(∃σ ∈ Σ)[*i* ∈ *R*_σ_]— and at most one label—again, mathematically expressed as $\left(\forall \sigma ,\sigma^{\prime }\in \Sigma \right)\left[R_{\sigma }\cap R_{\sigma^{\prime }}=\emptyset \right]$. Furthermore, in both theories every element must be ordered.

For example, the structure S=〈{1,2};∅,{1},{2},∅〉 is a case of a structure which is not interpretable as a string in either 𝔐^⊲^ or 𝔐^<^. Structure S in this case specifies two elements, one of which is labeled *a* and the other is labeled *b*, but the order of these elements remains unspecified. Another example of a structure which does not correspond to a string is S=〈{1};∅,{1},{1},∅〉; here there is one element which is labeled both a and b.

### 4.6. Subregular complexity

**Figure 4:**
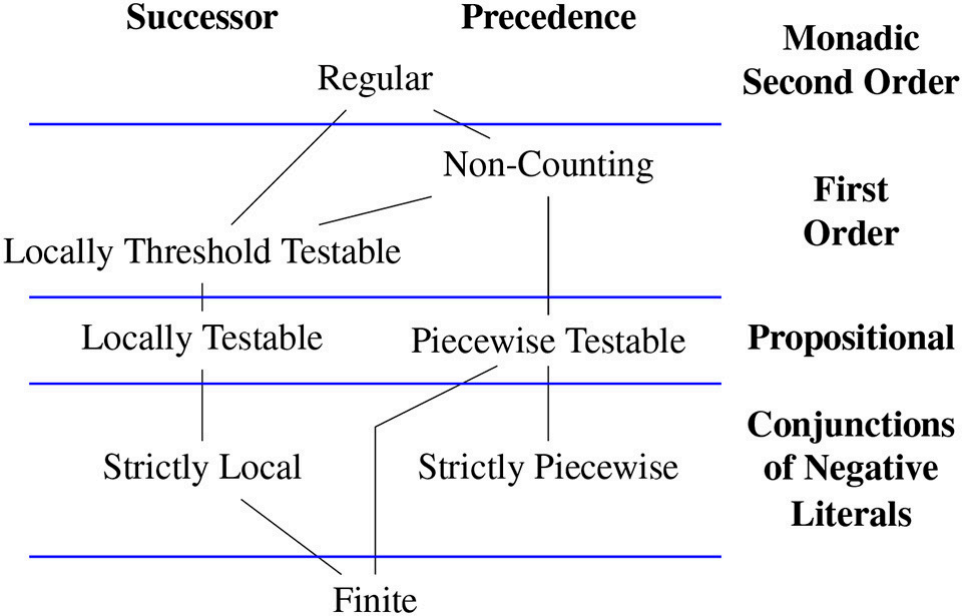
Subregular Hierarchies from a model-theoretic perspective.

Depending on the choice of model and logic different classes of stringsets arise (Büchi, 1960; McNaughton and Papert, 1971; Thomas, 1982, 1997; Rogers et al., 2010, 2013; Rogers and Pullum, 2011). Figure 4 shows proper inclusion relationships among many such classes. The figure and subsequent discussion provides logical characterizations for categorical stringsets, but stochastic versions for each can be given with weighted logics (Droste and Gastin, 2009). For completeness, language-theoretic definitions of each class are given below and other characterizations based on automata and algebra are omitted.

We have already defined regular stringsets as those characterized by a dfa or pdfa. Büchi (1960) showed these are exactly the categorical stringsets definable with weak mso logic with the order relation given as successor (or precedence, since the precedence relation is mso-definable from successor and vice versa). We now define the other classes in Figure 4 moving left-to-right and top-to-down.

DEFINITION 2 (Locally Threshold Testable Thomas, 1982).

A stringset *L* is Locally Threshold Testable iff there are two numbers *k* and *t* such that for all strings *u, v* ∈ Σ^*^ and *k*-factors $x\in {\text{factor}}_{k}\left(\left\{⋊\right\}\Sigma^{*}\left\{⋉\right\}\right)$, whenever *x* occurs either at least *t* times in both *u* and *v* or an equal number of times in both *u* and *v*, then either *u, v* ∈ *L* or *u, v* ∉ *L*.

In other words, membership of a string *w* in any LTT_*t, k*_ stringset is determined solely by the number of occurrences of each *k*-factor in *w*, counting them only up to some threshold *t*. Thomas (1982) showed that fo-definable categorical stringsets with the successor model 𝔐^⊲^ are exactly the Locally Threshold-Testable stringsets.

DEFINITION 3 (Non-Counting). A stringset *L* is Non-Counting iff there is a *k* such that for all *w, u, v* ∈ Σ^*^, if *wuv* ∈ *L* then *wu*^*k* + 1^*v* ∈ *L*.

McNaughton and Papert (1971) showed that fo-definable stringsets with the precedence model 𝔐^<^ are exactly the Non-Counting stringsets. They also prove languages in the Non-Counting class are exactly those definable with star-free generalized regular expressions and exactly those obtained by closing LT stringsets under concatenation. Hence this class also goes by the names “Star-Free” and “Locally Testable with Order.” The Non-Counting class properly includes the Locally Threshold Testable languages because the successor relation is fo-definable from precedence but not vice versa.

Finally, observe that stringsets that are regular but not Non-Counting typically count modulo some *n*. For example, the stringset which contains all and only strings with an even number of as is not Non-Counting, but regular.

DEFINITION 4 (Locally Testable Rogers and Pullum, 2011).

Language *L* is Locally *k*-Testable (LT_*k*_) iff there is some *k* such that, for all strings *x* and *y*, if factor_*k*_(⋊*x*⋉) = factor_*k*_(⋊*y*⋉) then *x* ∈ *L*↔*y* ∈ *L*. Stringset *L* is Locally Testable (LT) if there is some *k* such that *L* ∈ LT_*k*_.

From a logical perspective, Locally Testable languages are ones given by a propositional calculus whose propositions correspond to factors (Rogers and Pullum, 2011). With respect to fo logic, they may be understood as belonging to the *B*(Σ_1_), which is the Boolean closure of fo formulas (with successor) which begin with a single block of existential quantifiers in prenex normal form (Thomas, 1997). Note that LT_*k*_ class equals LTT_1,*k*_.

DEFINITION 5 (Piecewise Testable). A language *L* is Piecewise *k*-Testable (PT_*k*_) iff there is some *k* such that, for all strings *x* and *y*, if subseq_*k*_(*x*) = subseq_*k*_(*y*) then *x* ∈ *L*↔*y* ∈ *L*. Stringset *L* is Piecewise Testable (PT) if there is some *k* such that *L* ∈ PT_*k*_.

Piecewise Testable languages are ones given by a propositional calculus whose propositions correspond to subsequences (Rogers et al., 2013). With respect to fo logic, they may be understood as belonging to the *B*(Σ_1_), which is the set of Boolean closure of fo formulas (with precedence) which begin with a single block of existential quantifiers in prenex normal form (Thomas, 1997).

DEFINITION 6 (Strictly Local Rogers and Pullum, 2011). A stringset *L* is Strictly *k*-Local (SL_*k*_) iff whenever there is a string *x* of length *k*−1, and strings *u*_1_, *v*_1_, *u*_2_ and *v*_2_ such that *u*_1_*xv*_1_, *u*_2_*xv*_2_ ∈ *L*, then *u*_1_*xv*_2_ ∈ *L*. Stringset *L* is Strictly Local (SL) if *L* ∈ SL_*k*_ for some *k*. We say *L* is *closed under suffix substitution*.

From a logical perspective, Strictly *k*-Local languages are ones given by a conjunction of negative literals (propositions) where literals correspond to *k*-factors (Rogers and Pullum, 2011). This means that a Strictly *k*-Local stringset only includes strings which do not contain any forbidden substring of length *k* (of which there can only be finitely many). For example, the conjunction ¬*aa* ∧ ¬*bb* means that *aa* and *bb* are forbidden substrings. If Σ = {*a, b*} then the only strings satisfying this expression alternate *a*s and *b*s. With respect to fo logic, Strictly Local stringsets may be understood as belonging to Π_1_, which is the set of fo formulas (with successor) which begin with a single block of universal quantifiers in prenex normal form (Thomas, 1997).

From an automata perspective, the SL_*k*_ class of stringsets is represented by a rdfa as follows. The states are strings whose lengths are less than *k* (the start state corresponds to the empty string), and its δ function maps a state *q* and symbol σ ∈ Σ to the longest suffix of *qσ* whose length is less than *k*. For instance, if *k* = 4 then δ(*a, b*) = *ab* and δ(*abc, a*) = *bca*. With the structure of the rdfa so determined, each stringset S ∈ SL_*k*_ reduces to a particular functions ρ and α. Such dfas define categorical SL_*k*_ stringsets and such pdfas define stochastic ones. The experiments in Sections 7 and 9 have SL stringsets as learning targets.

DEFINITION 7 (Strictly Piecewise Rogers et al., 2010). A language *L* is Strictly *k*-Piecewise (SP_*k*_) iff subseq_*k*_(*w*) ⊆ subseq_*k*_(*L*) implies *w* ∈ *L*. Stringset *L* is Strictly Piecewise (SP) if there is a *k* such that it belongs to SP_*k*_; equivalently, *L* belongs to SP iff *L* is closed under subsequence.

From a logical perspective, Strictly Piecewise languages are ones given by a conjunction of negative propositions where propositions correspond to factors (Rogers et al., 2010). This means that a Strictly *k*-Piecewise stringset only includes strings which do not contain any forbidden subsequences of length *k* (of which there can only be finitely many). For example, the conjunction ¬*aa* ∧ ¬*bb* means that *aa* and *bb* are banned subsequences. If Σ = {*a, b, c*} then the only strings satisfying this expression contain at most one *a* and at most one *b*. With respect to fo logic, they may be understood as belonging to Π_1_, which is the set of fo formulas (with precedence) which begin with a single block of universal quantifiers in prenex normal form (Thomas, 1997). Rogers et al. (2010) provide an automata-theoretic characterization.

While the subregular classes of stringsets in the above diagram exhibit different properties, the logical characterizations make the parallels between the two sides of the hierarchy clear. The Strictly Local and Strictly Piecewise classes are relevant to the experiments presented later.

### 4.7. Sub-structures

For any two relational structures $S_{1}$ and $S_{2}$ of the same theory, we say $S_{1}$ is a *sub-structure* of $S_{2}$ (written $S_{1}⊑S_{2}$) iff there exists an injective homomorphism *h* which maps every element in *D*_1_, the domain of $S_{1}$, to elements in *D*_2_, the domain of $S_{2}$, such that all *n*-tuples of elements of *D*_1_ and for all *n*-ary relations *R*_*ij*_ with *i* ∈ {1, 2} and *j* = 1, …, *m*, we have (*x*_1_, …, *x*_*n*_) ∈ *R*_1*j*_ iff (*h*(*x*_1_), …*h*(*x*_*n*_)) ∈ *R*_2*j*_.

For example under 𝔐^⊲^, $M_{ab}$ is a sub-structure of $M_{cabb}$. (Let *h* map 1 to 2 and 2 to 3.) Under 𝔐^<^, $M_{cb}$ is a sub-structure of $M_{cabb}$. (Let *h* map 1 to 1 and 2 to 3 (or 4).)

The lemma below is not difficult to prove.

LEMMA 1. *For all u, v* ∈ Σ^*^, *word u is a substring of v iff* $M_{u}^{⊲}⊑M_{v}^{⊲}$. *Likewise, u is a subsequence of v iff* $M_{u}^{<}⊑M_{v}^{<}$.

Not only do these facts help make clear the similarities between substrings and subsequences observed in earlier works (Lothaire, 1997, 2005; García and Ruiz, 2004), they also show that what a sub-structure is depends on the model. As we will see in sections 8 and 9, sub-structures play an important role in unconventional models and relational learning, where it provides a generality relation in the sense of De Raedt (2008).

### 4.8. Learnability of subregular classes

From a learning perspective, the characterizations place limits on what kinds of stringsets can be learned when learning systems rely on fo logic. mlns, for example, can never exactly learn any regular stringset that is not Non-Counting because those cannot be expressed with fo formulas. On the other hand whether a mln can learn a Non-Counting stringset may well depend in part on whether the word model employs the successor relation, the precedence relation or both.

It is known that for given *k*, the strictly *k*-local stringsets are identifiable in the limit from positive data (García et al., 1990). This result can be generalized to the SP_*k*_, LT_*k*_, PT_*k*_, and LTT_*t,k*_ stringsets (García and Ruiz, 2004; Heinz, 2010; Heinz et al., 2012).

## 5. Unconventional models

In many domains of interest—including natural language processing and robotic planning and control—stringsets are used to characterize aspects of the nature of system. While conventional word models may be sufficiently expressive for problems in these domains, they do not take advantage of *domain-specific knowledge*. Specifically, in the conventional word models discussed previously, the unary relations are such that each position in a word can only satisfy one such relation. It is not the case that a position can satisfy two unary relations simultaneously.

Here is a simple motivating example. If we restrict ourselves to the alphabet {a, …z, A, …Z}, then under a conventional model there are 52 unary relations. Upper and lowercase versions of these symbols, e.g., a and A, are in no way associated. However, an unconventional word model that takes such associations into account might posit just 27 unary relations {a, …z, capital}. For a word like *Mama*, both capital(1) and m(1) would be true. In this way, this unconventional model captures the similarity between corresponding lowercase and uppercase letters.

In the context of learning stringsets, these correspondences can, and should be, exploited. Current learning approaches based on automata are challenging since automata are best understood as processing individual symbols. On the other hand, *relational* learning methods can immediately be applied to this problem. As explained in Section 4, different logical languages from different word models yield different classes of stringsets. The subregular hierarchies in Figure 4 exemplify the nature of the classes obtained when representational primitives are changed between successor and precedence models. The goal here is to expand the horizontal axis in Figure 4 to consider word models where the assumption that the unary relations are disjoint, is relaxed.

In the remainder of this paper we apply mlns (Section 3) to learning stringsets (Section 4) with model-theoretic treatments of words (Section 2). We present three experimental case studies. In each case study, we provide the formulas and learn the weights.

The first case study serves as a sanity check. We expect that mlns should be able to learn stringsets from examples, regardless of whether the strings are represented with conventional or unconventional word models. Therefore, we ask whether an mln can mimic *n-gram models*. These are parametric models widely used in natural language processing (nlp) which implicitly adopt the conventional successor model. From the perspective of the Subregular Hierachies (Figure 4), n-gram models are stochastic Strictly *n*-Local stringsets. The case study explains how to express the logic underlying SL_*k*_ stringsets, and how to express this logic with mlns. The experimental result shows that the learning behavior of the mln closely mimics the learning behavior of the n-gram model. We conclude that mlns can instantiate n-gram models, but are much more general because other parametric models can be instantiated with mlns by changing both the underlying model-theoretic representation of strings and the logical formulas.

This knowledge is put to use in the subsequent case studies. The second case study is about the problem of learning an aspect of one's phonological grammar: how to assign stress (a type of prominence) in words. The stress pattern we describe is amenable to multiple logical descriptions. We offer two: one using a conventional precedence model and one with an unconventional precedence model. We show learning the stress pattern requires less data and less computation time if the unconventional model is used.

The third case study illustrates the application of mlns and unconventional models to an engineering problem where one (a machine) has to learn how two pieces of hardware—in this case, mobile robots—can interact with each other and work together as a team. In the particular case study, the interaction is meaningful and needed, because the task that needs to be performed cannot be accomplished by only one robot working in isolation. The idea behind this case study is that one may obtain a set of example interaction cases by having a skilled (human) operator coordinating the robots over, possibly, a variety of different tasks. Then the problem is how to construct a formal model that captures and generalize possible ways of interaction between these agents, which would be an important first step into planning the coordination in a fully autonomous way at a later stage. In addition to demonstrating that the statistical relational learning framework is general enough to be useful in different application spaces, this last case study allows one to draw similar conclusions as before regarding the efficiency of learning algorithms when applied to unconventional models.

## 6. Implementation in Alchemy

We used the software package Alchemy 2 (Domingos and Lowd, 2009) to implement the experiments with mlns described in the following sections. Appendix A in Domingos and Lowd (2009) provides details of the Alchemy system. The Alchemy website http://alchemy.cs.washington.edu provides source code and additional documentation.

For each experiment, there are two input files for weight learning. One is a .mln file that lists the fo formulas in the language of the model-theoretic representation which define the mln.

Our case studies are mostly limited to Strictly *k*-Local and Strictly *k*-Piecewise stringsets so we illustrate how they can be implemented as mlns in the .mln files with a simple example. Let Σ = {*a, b*} and consider a Strictly 2-Local grammar. The input .mln file contains statements of the possible predicates in the successor model. Since there are four 2-factors, {*aa, ab, ba, bb*}, there would be four fo statements (where adjacent stands for the successor relation):

$$\begin{array}{l} \text{adjacent}\left(x,y\right)\wedge \text{a}\left(x\right)\wedge \text{a}\left(y\right)\\ \text{adjacent}\left(x,y\right)\wedge \text{a}\left(x\right)\wedge \text{b}\left(y\right)\\ \text{adjacent}\left(x,y\right)\wedge \text{b}\left(x\right)\wedge \text{a}\left(y\right)\\ \text{adjacent}\left(x,y\right)\wedge \text{b}\left(x\right)\wedge \text{b}\left(y\right) \end{array}$$

In the same .mln file, we would declare the following predicates:

$$\begin{array}{l} \text{adjacent}(\text{char},\text{char})\\ \text{a}(\text{char})\\ \text{b}(\text{char}) \end{array}$$

If we were to consider a Strictly 2-Piecewise grammar, then the .mln file would be very similar to the one just described. The only difference would be that instead of the adjacent predicate, the four fo formulas would contain the follows predicate (which stands for the precedence relation).

The other input file to Alchemy 2 is a training database (.db file) which provides the sample data which is the input to the mln. The training database provides a list of evidential predicates, also called *ground atoms*. These essentially are the model-theoretic representations of the strings in the data sample.

To represent each input string in the training database, each position in each string are indexed with a dummy denotation. We used capitalized letters of the alphabet and their combinations. These positions correspond to elements of the domain in a word model. Having that, we then list the properties of each position, and also the binary relations between positions.

For example, a string in the training dataset of the current example might be ‘*cabb*’. For such a string, we get the following list of atoms in the .db file (cf. Figure 3) under the successor model 𝔐^⊲^.

$$\begin{array}{l} \{\text{c}\left(A\right),\text{a}\left(B\right),\text{b}\left(C\right),\text{b}\left(D\right),\text{adjacent}\left(A,B\right),\\ \;\text{adjacent}\left(B,C\right),\text{adjacent}\left(C,D\right)\} \end{array}$$

In an unconventional word model, more than one unary relation may be listed for some node. How the set of strings for each training dataset was generated for each case study is described later.

When Alchemy 2 is run with these input files, it produces an output file which provides the learned weights for each statement in the .mln file. For running the weight-learning function, we used generalized weight-learning (command-line option −g), with no listing of non-evidence predicates and default parameters.

Each of our case studies required some specific treatment beyond the overall methods described above, which we discuss as appropriate in the subsequent sections.

## 7. Comparison of MLNs with n-Gram models

*N*-gram models are widely used in natural language processing (Jurafsky and Martin, 2008). An *n*-gram model can be understood as a pdfa whose underlying structure is Strictly *n*-Local.

This section shows that mlns can mimic n-gram models. Specifically, our experiments demonstrate that a trained bigram model and a trained mln behave similarly. We leave establishing the theoretical facts for future research.

### 7.1. Target stochastic stringset

The pdfa *A* in Figure 1 in section 4.3 exemplifies a n-gram model with *n* = 2 and Σ = {*a, b, c*}. As such, it represents a stochastic Strictly 2-Local stringset $f_{A}:\Sigma^{*}\to \left[0,1\right]$, and is the learning target in this section. As mentioned in Section 4.4, the pdfa *A* defines a class of stochastic stringsets of which *f*_*A*_ is one. Learning *f*_*A*_ comes down to learning the ρ and α functions. pdfa *A* has 16 parameters, all necessary to fully specify ρ and α. Table 1 summarizes these values.

**Table 1:** The parameter values of pdfa *A* of Figure 1.

***q***	**ρ(*q, a*)**	**ρ(*q, b*)**	**ρ(*q, c*)**	**α(*q*)**
start	0.3333	0.3333	0.3333	0
a	0.3000	0.2000	0.2000	0.3000
b	0.2000	0.3000	0.2000	0.3000
c	0.2000	0.2000	0.3000	0.3000

### 7.2. Learning algorithms

To see how well mlns can learn the stochastic stringset defined by *A*, we generated samples of words from *A* to use as training data.

We fed these training samples to two learning algorithms. One algorithm is the one mentioned in section 4, which uses the structure of *A* to find parameters that yield the mle with respect to the family of stochastic distributions that *A* defines (see de la Higuera, 2010 for details). The other algorithm takes a mln with formulas that are intended to mimic the structure of *A*, and finds the weights that produce the mle with respect to the family of stochastic distributions this mln defines.

In natural language processing, the first approach is usually implemented in a way that incorporates *smoothing* (Chen and Goodman, 1999); the latter refers to a variety methods that assign nonzero probability to all possible strings, in an attempt to yield better learning outcomes when the training data size is small. This option is not adopted here since the emphasis of the present analysis is not on performance on varying training data size, but rather on comparing qualitatively the performance of a mln with a conventional model to the standard method of obtaining the mle of a given pdfa.

The formulas in the mln included logical statements in the form given in Section 6 for Strictly 2-Local grammars, in addition to statements related the beginnings and endings of words. We assumed that predicates initial and final can only occur on word-edges. Therefore, this mln was developed with signature 〈𝔇; ⊲, *R*_*a*_, *R*_*b*_, *R*_*c*_, *R*_initial_, *R*_final_〉. As such, the mln contained 16 fo statements, each of which correspond to a parameter of *A*. The complete .mln file is given in the Appendix.

### 7.3. Evaluation method

The models output by these learning algorithms were compared in two ways: by comparing the probabilities of subsquent symbols directly in the trained models and by calculating the perplexity the models give to a test set.

For the first comparison, we converted the weights obtained in the mln model into interpretable parameter values for *A*. Generally, whenever analyzing mlns, one should avoid the computation of the partition function *Z* through (1) whenever possible. One way for doing that is to find sets of conditional events that are mutually exclusive and collectively exhaustive (sum to one), and then look at the ratio of the probabilities of those conditional events.

For instance, suppose we are given two constants *x* and *y*; then P(b(*y*)|a(*x*), adjacent(*x, y*)) corresponds to ρ(*a, b*). We denote $S_{ab}$ the world in which a(*x*) = 1, b(*y*) = 1, adjacent(*x, y*) = 1, and zero is assigned to all other ground atoms. It follows that

$$\begin{array}{l} \text{P}\left(S_{ab}\right)=\frac{\rho \left(a,b\right)}{\text{P}(\text{a}\left(x\right),\text{adjacent}\left(x,y\right))} \end{array}$$

Let $F_{\sigma \sigma^{\prime }}=\text{adjacent}\left(x,y\right)\wedge \sigma \left(x\right)\wedge \sigma^{\prime }\left(y\right)$ and denote $w_{\sigma \sigma^{\prime }}$ its weight. Let σ range over the predicates {a, b, c, initial}, and σ′ range over {a, b, c, final}. Then let $S_{\sigma \sigma^{\prime }}$ be the structure of size two, for which $F_{\sigma \sigma^{\prime }}$ is true. According to Equation (3), the probability that the mln assigns to $S_{\sigma \sigma^{\prime }}$ is

$$\begin{array}{l} \text{P}\left(S_{\sigma \sigma^{\prime }}\right)=\frac{\text{exp}\sum_{\sigma \sigma^{\prime }}w_{i}n_{F_{\sigma \sigma^{\prime }}}\left(S_{\sigma \sigma^{\prime }}\right)}{Z} \end{array}$$

We want to determine ρ(*a, a*), ρ(*a, b*), ρ(*a, c*), and ρ(*a*, ⋉). These must sum to one. Observe that the ratio $\frac{\rho \left(a,a\right)}{\rho \left(a,b\right)}=\frac{\text{P}\left(S_{aa}\right)}{\text{P}\left(S_{ab}\right)}$ and

(4) $$\begin{array}{l} \frac{\text{P}\left(S_{aa}\right)}{\text{P}\left(S_{ab}\right)}=\frac{\frac{1}{Z}\text{exp}(\sum_{\sigma \sigma^{\prime }}w_{aa}n_{F_{aa}}\left(S_{aa}\right))}{\frac{1}{Z}\text{exp}(\sum_{\sigma \sigma^{\prime }}w_{ab}n_{F_{ab}}\left(S_{ab}\right))}\\ =\frac{\text{exp}(\sum_{\sigma \sigma^{\prime }}w_{aa}n_{F_{aa}}\left(S_{aa}\right))}{\text{exp}(\sum_{\sigma \sigma^{\prime }}w_{ab}n_{F_{ab}}\left(S_{ab}\right))} \end{array}$$

Notice that $S_{aa}$ has one true grounding only in *F*_*aa*_, and zero true grounding in all other formulas. Similarly, the world $S_{ab}$ has one true grounding only in *F*_*ab*_ and zero true grounding in all other formulas. Consequently, the ratio of the probabilities equals the ratio of the exponential of the weights of corresponding satisfied formulas, namely

$$\begin{array}{l} \frac{\rho \left(a,a\right)}{\rho \left(a,b\right)}=\frac{\text{P}\left(S_{aa}\right)}{\text{P}\left(S_{ab}\right)}=\frac{\text{exp}(\sum_{\sigma \sigma^{\prime }}w_{aa}n_{F_{aa}}\left(S_{aa}\right))}{\text{exp}(\sum_{\sigma \sigma^{\prime }}w_{ab}n_{F_{ab}}\left(S_{ab}\right))}=\frac{\text{exp}\left(w_{aa}\right)}{\text{exp}\left(w_{ab}\right)} \end{array}$$

Thus ρ(*a, a*) is expressed directly in terms of ρ(*a, b*). Calculating all such ratios and considering the fact that ρ(*a, a*) + ρ(*a, b*) + ρ(*a, c*) + ρ(*a*, ⋉) = 1 provides a solvable system of equations.

The second method examined the perplexity of a data set. Perplexity is a measure of model performance utilized in natural language processing (Jurafsky and Martin, 2008). It is an information theoretic measure of how well a model predicts the next symbol given the previous symbols. If *P*_*M*_(σ_*i*_∣σ_1_, …, σ_*i*−1_) denotes the probability that model *M* assigns to the *i*th symbol given the previous *i*−1 symbols in the string, then the perplexity of *M* is given by

(5) $$\begin{array}{l} 2^{-\frac{1}{n}\Sigma_{i=1}^{n}{\text{log}}_{2}P_{M}\left(\sigma_{i}|\sigma_{1},\ldots ,\sigma_{i-1}\right)} \end{array}$$

Low perplexity is an indication of model prediction accuracy.

### 7.4. Training data

The training data was randomly generated with the pdfa *A* in Figure 1. In other words, strings were drawn i.i.d. according to the probability distribution over Σ^*^ that *A* represents, which is the standard procedure for generating training data for learning pdfas (de la Higuera, 2010; Sicco Verwer and Eyraud, 2014). We considered three different sizes of training data: 20, 50, and 100 strings. We generated training data of different sizes because we are also interested in performance on small data sets. For each size, we generated 10 different datasets so we could aggregate results across them. As we will see in the next section, we found that this range of sizes in the training data was sufficient to show that MLEs and trained MLNs behave similarly, especially with 100 strings in the training data set.

Before training the mln, each training set had to be translated to a knowledge database (see Section 6). This entailed listing successor relations between adjacent nodes, along with the labels of the nodes. The strings in the training set were augmented with *initial* and *final* positions, so that a string *abc* was represented as

$$\begin{array}{l} \{\text{initial}\left(A\right),\text{a}\left(B\right),\text{b}\left(C\right),\text{c}\left(D\right),\text{final}\left(E\right),\\ \text{adjacent}\left(A,B\right),\text{adjacent}\left(B,C\right),\text{adjacent}\left(C,D\right),\\ \text{adjacent}\left(D,E\right)\} \end{array}$$

### 7.5. Results and discussion

Table 2 summarizes the results for each training sample of size *N*. The parameter values shown are averages obtained from randomly generating 10 samples of size *N* and running the learning algorithms on each sample. After each run of a learning algorithm, a test set of 10 test strings was generated by *A* and the perplexity of the learned model was calculated. This was done 1,000 times and these perplexity values were averaged.

**Table 2:** Mean parameter values and perplexity obtained by the two learning algorithms on the training sets. Standard deviations are shown in parentheses.

***q***	**Maximum likelihood estimates**				**MLN probabilities**			
	**ρ(*q, a*)**	**ρ(*q, a*)**	***ρ(q, c)***	**ρ(*q*, end)**	**ρ(*q, a*)**	**ρ(*q, a*)**	***ρ(q, c)***	**ρ(*q*, end)**
**20 STRING TRAINING SET**								
Start	0.3550	0.3150	0.3300	0	0.3433	0.2776	0.3790	9.5e-5
a	0.2532	0.2278	0.1914	0.3276	0.3078	0.2028	0.2335	0.2560
b	0.2354	0.3146	0.1418	0.3082	0.2643	0.3568	0.1596	0.2193
c	0.1717	0.2202	0.2664	0.3417	0.1467	0.2360	0.3614	0.2259
Perplexity	2,012.9 (1184.2)				1,794.1 (966.9)			
**50 STRING TRAINING SET**								
Start	0.3220	0.3360	0.3420	0	0.3288	0.3394	0.3318	3.0e-5
a	0.2914	0.1989	0.2101	0.2996	0.3588	0.1950	0.1913	0.2549
b	0.1949	0.2815	0.2267	0.2970	0.1930	0.3262	0.2250	0.2559
c	0.2040	0.2200	0.3009	0.2751	0.2097	0.2215	0.3382	0.2307
Perplexity	1,119.4 (272.9)				1,090.9 (192.8)			
**100 STRING TRAINING SET**								
Start	0.3190	0.3590	0.3220	0	0.3279	0.3466	0.3255	2.3e-5
a	0.2751	0.2214	0.1918	0.3118	0.3406	0.2120	0.1909	0.2565
b	0.1961	0.3008	0.2047	0.2985	0.1999	0.3442	0.2101	0.2457
c	0.2046	0.2057	0.2866	0.3030	0.2151	0.1961	0.3440	0.2449
		

The results of Table 2 confirm that a mln with formulas that instantiate the logical structure of a Strictly 2-Local stringset behaves similarly to a bigram model. The parameter values and perplexity scores across the two models are similar. In fact, the trained mln behaves like a smoothed bigram model since every parameter has nonzero values. This is likely due to the Gaussian prior used for the weights.

Thus, mlns can mimic the behavior of standard language models. The next two sections compare the effects of different representations on learning, by studying two mlns trained on the same data sets. The formulas in one mln are based on a conventional word model, and the formulas in the other are based on an unconventional word model. Since the only difference between the mlns is due to the nature of the word models, any differences observed in learning outcomes can reasonably be attributed to representation.

## 8. Unbounded stress patterns

This case study compares conventional and unconventional word models in light of the problem of phonological well-formedness. It is widely accepted in phonology that in many languages the syllables of a word have different levels of prominence, evident either from acoustic cues or perceptual judgments (Chomsky and Halle, 1968; Liberman, 1975; Schane, 1979; Hayes, 1995). For example, native English speakers generally agree that the second syllable in *America* stands out from the rest. This type of prominence is called *stress*.

The position of stress in a word is predictable in many languages, and a variety of stress patterns have been described (van der Hulst et al., 2010). Learning where stress falls is therefore a problem for children acquiring their native language, for second-language learners, and for many applications, including speech synthesis and recognition.

Predictable stress patterns can be broadly divided into two categories: bounded and unbounded. In *bounded* patterns, the position of stress is always within some fixed distance of the beginning or end of the word. Thus all bounded patterns are SL_*k*_ where *k* is the number of positions from the stressed syllable to the left or right word edge. *Unbounded* stress patterns are not bounded.

In some languages, an important factor for predicting stress is *syllable weight*. Put simply, syllable weight is determined by the length of the syllable and the number of different sounds included at the end of the syllable. Usually only two weights are distinguished: light (L) and heavy (H).^2^ Stress patterns that take syllable weight into account are *quantity-sensitive*. We focus on one such pattern, called Leftmost-Heavy-Otherwise-Rightmost (LHOR).

### 8.1. The LHOR stress pattern

Hayes (1995) describes four types of simple quantity-sensitive unbounded stress patterns. The pattern we study is exemplified by Kwak'wala, an indigenous language spoken on the Pacific Northwest Coast (Bach, 1975). Stress in Kwak'wala generally falls on the leftmost heavy syllable of the word. If the word has no heavy syllables, then stress falls on the rightmost light syllable. This pattern is therefore abbreviated LHOR (Leftmost-Heavy-Otherwise-Rightmost).

Let Σ = {L,H,Ĺ,$\overset{\prime }{\text{H}}$}. The acute accent denotes stress and *L* and *H* denote light and heavy syllables (so $\overset{\prime }{\text{H}}$ denotes a stressed heavy syllable). Let $L_{\text{LHOR}}$ be the set of all strings that obey the LHOR pattern. These are called *well-formed* words. Some examples are given in Table 3.

**Table 3:** Some well-formed words in $L_{\text{LHOR}}$.

Ĺ	LĹ	LLĹ	LLLĹ	LL$\overset{\prime }{\text{H}}$
$\overset{\prime }{\text{H}}$	$\overset{\prime }{\text{H}}$H	$\overset{\prime }{\text{H}}$LL	$\overset{\prime }{\text{H}}$HH	L$\overset{\prime }{\text{H}}$LHL

The dfa *A*_LHOR_ in Figure 2 computes the stringset $L_{LHOR}$; that is, it accepts all and only those strings which obey the LHOR pattern.

The well-formedness of a word in LHOR can be analyzed in terms of its subsequences of size 2 or smaller. The permissible and forbidden 2-subsequences in LHOR are shown in Table 4. If a word contains a single stressed syllable and does not contain any of the forbidden 2-subsequences, then it is well-formed.

**Table 4:** 2-subsequences in LHOR (Strother-Garcia et al., 2016, Table 2).

Permissible				Forbidden			
LL	HH	LĹ	HL	H$\overset{\prime }{\text{H}}$	ĹL	HĹ	ĹĹ
LH	$\overset{\prime }{\text{H}}$H	L$\overset{\prime }{\text{H}}$	$\overset{\prime }{\text{H}}$L	$\overset{\prime }{\text{H}}$$\overset{\prime }{\text{H}}$	ĹH	$\overset{\prime }{\text{H}}$Ĺ	Ĺ$\overset{\prime }{\text{H}}$

Heinz (2014) analyzes simple unbounded stress patterns like the one above and shows that they are neither SL nor SP. LHOR cannot be SL because it is not closed under suffix substitution. While both $\overset{\prime }{\text{H}}$L^*k*^L and L^*k*^Ĺ belong to $L_{\text{LHOR}}$, $\overset{\prime }{\text{H}}$L^*k*^Ĺ does not. LHOR is therefore not SL for any *k*. Moreover, it cannot be SP because it is not closed under subsequence. LL^*k*^Ĺ belongs to $L_{\text{LHOR}}$ but the subsequence LL does not, so LHOR is not SP_*k*_ for any *k*.

Furthermore, Heinz (2014) shows that LHOR and similar patterns can be understood as the intersection of two stringsets: a Strictly 2-Piecewise one which bans the forbidden 2-subsequences and a Locally 1-Testable one which requires words to contain a stress.^3^ This analysis of LHOR extends similarly for other simple unbounded stress patterns.

### 8.2. Logical characterizations of LHOR

Strother-Garcia et al. (2016) provide two logical characterizations of the LHOR pattern. One is based on a conventional word model and the other on an unconventional one. Of interest is the reduction in complexity of the logical formulas when the unconventional word model is adopted.

Consider the conventional Precedence Word Model 𝔐^<^ (Section 4.5) with Σ = {L, H, Ĺ, $\overset{\prime }{\text{H}}$}. The signature of 𝔐^<^ is thus 〈*D*; <, *R*_L_, *R*_H_, *R*_Ĺ_, *R*_$\overset{\prime }{\text{H}}$_〉. The LHOR pattern can be defined with formula templates *F* and *G*. Letting *a, b* range over Σ, we define

$$\begin{array}{l} F_{ab}=\left(\exists x,y\right)\left[x<y\wedge R_{\text{a}}\left(x\right)\wedge R_{\text{b}}\left(y\right)\right]\\ G_{a}=\left(\exists x\right)\left[R_{\text{a}}\left(x\right)\right] \end{array}$$

For example, strings that satisfy *F*_H$\overset{\prime }{\text{H}}$_ contain the 2-subsequence H$\overset{\prime }{\text{H}}$ and strings that satisfy *G*_$\overset{\prime }{\text{H}}$_ contain the symbol $\overset{\prime }{\text{H}}$. The set of banned subsequences in LHOR (Table 4) is *B* = {H$\overset{\prime }{\text{H}}$, $\overset{\prime }{\text{H}}$$\overset{\prime }{\text{H}}$, ĹL, ĹH, HĹ, $\overset{\prime }{\text{H}}$Ĺ, HĹ, ĹĹ, Ĺ$\overset{\prime }{\text{H}}$}. Then

$$\begin{array}{l} \varphi_{\text{LHOR}}\;\underline{\underline{\text{def}}}\;\underset{v\in B}{\wedge }\neg F_{v}\wedge \left(G_{\text{Ĺ}}\vee G_{\overset{\prime }{\text{H}}}\right) \end{array}$$

is true of string *w* iff *w* contains no member of *B* as a subsequence and it contains either Ĺ or $\overset{\prime }{\text{H}}$. Formula φ_LHOR_ is in 2-conjunctive normal form (cnf).

$L_{\text{LHOR}}$ is the set of all strings *w* whose models $M_{w}$ satisfy φ_LHOR_.

$$\begin{array}{l} L_{\text{LHOR}}=\;\{w\in \Sigma^{*}|M_{w}⊨\varphi_{\text{LHOR}}\} \end{array}$$

The unconventional word model 𝔐 is similar to 𝔐^<^ with an important caveat: each domain element may belong to more than one unary relation. In other words, each position may bear multiple labels. Let Σ′ = {light, heavy, stress}. Then 𝔐 includes the unary relations *R*_L_, *R*_H_, *R*_S_ for light, heavy, and stress, respectively. The elements of Σ′ can be interpreted in terms of the conventional alphabet as follows:

$$\begin{array}{l} R_{\text{L}}(x)=\left\{x\in \left\{\text{L},\overset{\prime }{\text{L}}\right\}\right\}\\ R_{\text{H}}(x)=\left\{x\in \left\{\text{H},\overset{\prime }{\text{H}}\right\}\right\}\\ R_{\text{S}}(x)=\left\{x\in \left\{\overset{\prime }{\text{L}},\overset{\prime }{\text{H}}\right\}\right\} \end{array}$$

For example, if position *x* in a string is labeled $\overset{\prime }{\text{H}}$, both *R*_H_(*x*) and *R*_S_(*x*) are true in the unconventional model. The symbol $\overset{\prime }{\text{H}}$ is now a shorthand for stress and heavy. As in the case of capital and lowercase letters (Section 5), both models are used to represent the same objects (members of $L_{\text{LHOR}}$). The unconventional model 𝔐 captures an important linguistic generalization that is not apparent in the conventional model: that stress is related to, but separable from, syllable weight.

The unconventional model provides a richer array of sub-structures (section 4.7) with which generalizations can be stated. Given 𝔐 and Σ′, Table 5 shows the possible sub-structures of size one, taking into account that syllables cannot be both light and heavy. The table also provides a symbol we use in this text to represent each possibility. Thus $\overset{\prime }{\text{H}}$ and Ĺ are fully-specified structures, while H and L represent heavy and light syllables that are unspecified for stress. Similarly, $\overset{´}{\sigma }$ represents a stressed syllable unspecified for weight, and σ is a completely unspecified syllable.

**Table 5:** Feature geometry for LHOR sub-structures of size 1.

**Symbol**	**Features**
$\overset{\prime }{\text{H}}$	heavy(*x*) ∧ stress(*x*)
Ĺ	light(*x*) ∧ stress(*x*)
H	heavy(*x*)
L	light(*x*)
$\overset{´}{\sigma }$	stress(*x*)
σ	∅

Strother-Garcia et al. (2016) construct a new formula under 𝔐 that also describes LHOR exactly. Recall that every word must have at least one stressed syllable. Under 𝔐, the formula representing this fact is $G_{\overset{´}{\sigma }}$. This structure is *underspecified*; it models no word in $L_{\text{LHOR}}$, but is a sub-structure of both $M_{\overset{\prime }{\text{H}}}$ and $M_{\text{Ĺ}}$.

The banned sub-structures are also simplified under 𝔐. Recall here that a stressed light is only permissible if it is the final syllable. Thus one of the banned sub-structures in the LHOR pattern is a stressed light followed by any other syllable, given by the formula *F*_Ĺσ_. Again, this structure is underspecified. It is a sub-structure of four of the forbidden 2-subsequences in Table 4: ĹH, Ĺ$\overset{\prime }{\text{H}}$, ĹL, and ĹĹ.

In a word with one or more heavy syllables, the stress must fall on the leftmost heavy. Consequently, a heavy syllable may not be followed by any stressed syllable. This is represented by the formula $F_{\text{H}\overset{´}{\sigma }}$, which is a sub-structure of the remaining four forbidden 2-subsequences from Table 4: H$\overset{\prime }{\text{H}}$, $\overset{\prime }{\text{H}}$$\overset{\prime }{\text{H}}$, HĹ, and $\overset{\prime }{\text{H}}$Ĺ.

Thus, LHOR can be described with a 1-CNF formula under 𝔐,

$$\begin{array}{l} \psi_{\text{LHOR}}=F_{\overset{´}{\sigma }}\wedge \neg F_{\text{Ĺ}\sigma }\wedge \neg F_{\text{H}\overset{´}{\sigma }} \end{array}$$

which contrasts with the 2-CNF formula φ_LHOR_ under 𝔐^<^.

Formula ψ_LHOR_ refers to sub-structures of size 2 or less, which are analogous to 2- and 1-subsequences. The unconventional word model permits a statement of the core linguistic generalizations of LHOR without referring to a seemingly arbitrary list of subsequences.

Strother-Garcia et al. (2016) point out that 1-CNF formulas are known to be learnable with less time and data than 2-CNF formulas (Valiant, 1984). They also demonstrate an algorithm that learns 1-CNF formulas exactly. The next sections compare how well mlns can learn the LHOR pattern with the conventional and unconventional word models.

### 8.3. Markov logic networks with conventional and unconventional models

Here we describe the two mlns used in this experiment. To illustrate the differences between the conventional and unconventional models, Table 6 shows how the word L$\overset{\prime }{\text{H}}$L would be represented in the database files in Alchemy.

**Table 6:** Conventional and unconventional word models for L$\overset{\prime }{\text{H}}$L.

**Conventional**	**Unconventional**
L(*A*)	L(*A*)
$\overset{\prime }{\text{H}}$(*B*)	H(*B*), stress(*B*)
L(*C*)	L(*C*)
follows(*A, B*)	follows(*A, B*)
follows(*A, C*)	follows(*A, C*)
follows(*B, C*)	follows(*B, C*)

The different word models also determined a different set of formulas in each of the mlns. For the conventional word model, all possible formulas of the form *F*_*ab*_ = adjacent(*x, y*) ∧ a(*x*) ∧ b(*y*) with a, b ∈ {L,H,Ĺ,$\overset{\prime }{\text{H}}$} were included, as explained in Section 7. This yields 16 statements.

For the mln with the unconventional model, if all possible formulas with two variables of the form *F*_*ab*_ with a, b belonging to the six sub-structures shown in Table 5 were included then there would be 36 statements. This increase occurs because positions in a string may satisfy more than one predicate.

However, we do not think it is appropriate to include them all. A sentence of the form “*x* < *y* ∧ *P*(*x*) ∧ *Q*(*y*)” can be interpreted as a prediction that position *y* has property *Q* given that position *x* with property *P* precedes it. There are three properties of interest for position *y*: heavy, light, or stressed. With respect to the predictor (the *x* position), we are interested in how individual properties (heavy, light, stressed) and how possible combinations of properties (of which there are two, heavy-stressed and light-stressed) predict the properties of *y*. For these reason, we only included formulas that predict one atomic property of *y* given the properties that may hold of position *x*. In other words the mln included formulas *F*_*ab*_ = adjacent(*x, y*) ∧ a(*x*) ∧ b(*y*) with a ∈ {L,H,$\overset{´}{\sigma }$,Ĺ,$\overset{\prime }{\text{H}}$} and $\text{b}\in \left\{L,H,\overset{´}{\sigma }\right\}$ where these symbols are a shorthand for the logical expressions shown in Table 5. This yielded 15 statements.

As mentioned, the LHOR pattern also *requires* sub-structures as indicated with formulas of type *G*_*a*_. Thus for both the conventional and unconventional word models, we also included statements which require sub-structures in strings. Our initial efforts in this regard failed because Alchemy quickly runs out of memory as it converts all existential quantification into a CNF formula over all the constants in the database file. To overcome this hurdle, we instead introduced statements into the database file *about* each string. This work-around essentially encoded the information in the existential formula as a property of another constant in the database. If there were *n* strings in the database file, we included constants *S*_1_, *S*_2_, …*S*_*n*_ which represented each string. In the conventional model, predicates isString(*x*), hasLstr(*x*), and hasHstr(*x*) were included. These predicates declare that *x* is a string, *x* contains a light and stressed syllable, and *x* contains a heavy and stressed syllable, respectively. The mln included the formula isString(*x*) ∧ (hasHstr(*x*)∨hasLstr(*x*)). In the unconventional word model, predicates isString(*x*) and hasStress(*x*) were included, where hasStress(*x*) states that string *x* has a stressed syllable. The mln included the formula isString(*x*) ∧ hasStress(*x*).

The .mln files of both the conventional and unconventional model for the stress example can be found in the Appendix.

### 8.4. Training data

We generated data sets in six sizes: 5, 10, 20, 50, 100, and 250 strings. For each size, we generated ten different datasets. We generated training data of different sizes because we were also interested in how well the mlns generalized from small data sets.

To generate a training data set, we first randomly generated strings from length one to five inclusive from the alphabet Σ = *{H, L}*. As a second step, we assigned stress to the correct syllable based on the LHOR pattern. These strings were then translated into a training database file for the mln based on its word model.

Table 7 reports the runtime of the weight-learning algorithm for both mlns with the conventional and unconventional models, over 5, 10, 20, 50, 100, and 250 strings. Unsurprisingly, the runtime for the unconventional models was slightly shorter than for the conventional models, since the unconventional models contained one less statement.

**Table 7:** Runtime of learning weights for linguistic statements.

	**Conventional model**	**Unconventional model**
5 strings	0.29s	0.27s
10 strings	0.51s	0.51s
20 strings	1.94s	1.89s
50 strings	10.28s	10.66s
100 strings	58.76s	49.43s
250 strings	8 min, 36.02s	7 min, 57.48s

### 8.5. Evaluation method

Two types of evaluations were conducted to address two questions. Did the mlns plausibly learn the LHOR pattern and how much data was necessary to learn it?

First, to evaluate whether the mlns correctly identified the LHOR pattern, we conducted an analysis of the trained models. Similar to Section 7, we find conditional events whose probabilities sum to one, identify their ratios, and solve for the probabilities of four structures which represent the generalizations of interest. These generalizations are shown below (cf. ψ_LHOR_).

(G1) No syllables follow stressed light syllables.
(G2) No stressed syllable follows a heavy syllable.
(G3) There is at least one stressed syllable.
(G4) There is at most one stressed syllable.

We elaborate on the analysis for G2; the analyses for the rest is similar. For the conventional model, let two constants (positions) *A* and *B* be given, and consider the following conditional probabilities.

$$\begin{array}{l} {\text{P}}_{1}=\text{P}(\overset{´}{\text{H}}\left(B\right)|\text{H}\left(A\right),\text{follows}\left(A,B\right))\\ {\text{P}}_{2}=\text{P}(\overset{´}{\text{L}}\left(B\right)|\text{H}\left(A\right),\text{follows}\left(A,B\right))\\ {\text{P}}_{3}=\text{P}(\text{L}\left(B\right)|\text{H}\left(A\right),\text{follows}\left(A,B\right))\\ {\text{P}}_{4}=\text{P}(\text{H}\left(B\right)|\text{H}\left(A\right),\text{follows}\left(A,B\right)) \end{array}$$

These probabilities are all disjoint and sum to one i.e., *P*_1_ + *P*_2_ + *P*_3_ + *P*_4_ = 1. The probability of P_1_ is given explicitly as

$$\begin{array}{l} {\text{P}}_{1}=\frac{\text{P}(\overset{´}{\text{H}}\left(B\right),\text{H}\left(A\right),\text{follows}\left(A,B\right))}{\text{P}(\text{H}\left(A\right),\text{follows}\left(A,B\right))} \end{array}$$

Probabilities P_2_, P_3_, and P_4_ are calculated similarly. Let ${\text{P}}_{1}^{S_{1}}=\text{P}(\text{H}\left(A\right),\text{follows}\left(A,B\right))$; this is the probability of the world $S_{1}$:

$$\begin{array}{c} S_{1}=\{\text{H}\left(A\right)\wedge \text{follows}\left(A,B\right)\wedge \overset{´}{\text{H}}\left(B\right)\wedge \neg \overset{´}{\text{L}}\left(A\right)\wedge \neg \overset{´}{\text{H}}\left(A\right)\wedge \neg \text{H}\left(B\right)\\ \wedge \neg \text{L}\left(B\right)\wedge \neg \text{L}\left(A\right)\wedge \neg \overset{´}{\text{L}}\left(B\right),\neg \text{follows}\left(B,A\right)\} \end{array}$$

Worlds *S*_2_, *S*_3_, *S*_4_ (with probabilities ${\text{P}}_{2}^{S_{2}}$, ${\text{P}}_{3}^{S_{3}}$, ${\text{P}}_{4}^{S_{4}}$) can be defined for *P*_2_, *P*_3_, *P*_4_ respectively, in the similar way. Given two syllables, it is obvious that *P*_1_ + *P*_2_ is the probability that a stressed syllable comes after H(*A*), which we denote $\text{P}\left(\text{H}\overset{´}{\sigma }\right)$. Likewise, *P*_3_ + *P*_4_ is the probability that an unstressed syllable comes after H(*A*), which we denote $\text{P}\left(\text{H}\neg \overset{´}{\sigma }\right)$. We also know $\text{P}\left(\text{H}\overset{´}{\sigma }\right)+\text{P}\left(\text{H}\neg \overset{´}{\sigma }\right)=1$. Thus we can compare $\text{P}\left(\text{H}\overset{´}{\sigma }\right)$ and $\text{P}\left(\text{H}\neg \overset{´}{\sigma }\right)$, using *N* to denote the number of formulas, *w*_*i*_ the weight for formula *F*_*i*_, and $n_{i}\left(S_{i}\right)$ the number of true groundings of formula *F*_*i*_ in world $S_{i}$:

$$\begin{array}{l} \frac{\text{P}\left(\text{H}\overset{´}{\sigma }\right)}{\text{P}\left(\text{H}\neg \overset{´}{\sigma }\right)}=\frac{{\text{P}}_{1}^{S_{1}}+{\text{P}}_{2}^{S_{2}}}{{\text{P}}_{3}^{S_{3}}+{\text{P}}_{4}^{S_{4}}}=\frac{\sum_{j=1}^{2}\text{exp}\left(\frac{\sum_{i=1}^{N}w_{i}n_{i}\left(S_{j}\right)}{Z}\right)}{\sum_{j=3}^{4}\text{exp}\left(\frac{\sum_{i=1}^{N}w_{i}n_{i}\left(S_{j}\right)}{Z}\right)}\\ =\frac{\sum_{j=1}^{2}\text{exp}\left(\sum_{i=1}^{N}w_{i}n_{i}\left(S_{j}\right)\right)}{\sum_{j=3}^{4}\text{exp}\left(\sum_{i=1}^{N}w_{i}n_{i}\left(S_{j}\right)\right)} \end{array}$$

The closer this ratio is to zero, the higher the confidence on the statement that the mln has learned (G2) that “No stressed syllable follows a heavy syllable.”

The analysis of the mlns based on both the conventional and unconventional models proceeds similarly.

Our second evaluation asked how much training is needed for each model to reliably learn the generalizations. Here we tested both models on small training samples. Specifically, we conducted training and analysis on 10 sets of 10 training examples and 10 sets of 5 training examples.

Prior to running the models, we arbitrarily set a threshold of 0.05. If the ratios calculated with the weights of the trained model were under this threshold, we concluded the model acquired the generalizations successfully. Otherwise, we concluded it failed. We then measured the proportion of training sets on which the models succeeded.

### 8.6. Results

Given a training sample with 100 examples, the resultant ratios representing each generalization for the mlns instantiating the conventional and unconventional word models are presented in Table 8. Three ratios are presented for G4 because two stressed syllables can occur in one of four ways: a stressed syllable follows $\overset{\prime }{\text{H}}$, a stressed syllable precedes $\overset{\prime }{\text{H}}$, a stressed syllable precedes Ĺ, or a stressed syllable follows Ĺ. The last case is already included in G1 (No syllable follows Ĺ), so the other three ratios are presented.

**Table 8:** Summary of ratios from one training sample with 100 examples.

**Generalization**	**Ratio**	**Conventional model**	**Unconventional model**
(G1)	$\frac{\text{P}\left(\text{Ĺ}\sigma \right)}{\text{P}\left(\sigma \text{Ĺ}\right)}$	0.0193	6e-6
(G2)	$\frac{\text{P}\left(\text{H}\overset{´}{\sigma }\right)}{\text{P}\left(\text{H}\neg \overset{´}{\sigma }\right)}$	0.1030	7e-4
(G3)	$\frac{\text{P}\left(\exists \overset{´}{\sigma }\right)}{\text{P}\left(\neg \exists \overset{´}{\sigma }\right)}$	4e-6	0.0185
(G4)	$\frac{\text{P}\left(\overset{´}{\text{H}}\overset{´}{\sigma }\right)}{\text{P}\left(\overset{´}{\text{H}}\neg \overset{´}{\sigma }\right)}$	7.6e-6	6.4e-10
(G4)	$\frac{\text{P}\left(\overset{´}{\sigma }\overset{´}{\text{H}}\right)}{\text{P}\left(\neg \overset{´}{\sigma }\overset{´}{\text{H}}\right)}$	0.0013	5e-10
(G4)	$\frac{\text{P}\left(\overset{´}{\sigma }\overset{´}{\text{L}}\right)}{\text{P}\left(\neg \overset{´}{\sigma }\overset{´}{\text{L}}\right)}$	0.0023	5.7e-10

*The closer the ratio is to zero, the better the generalization*.

Both mlns assign small values to these ratios, which indicate that they successfully learned the generalizations given 100 training examples. However, in most cases, the unconventional model generalized better.

With respect to the question of how much data was required to learn the LHOR pattern, we conclude that mlns using unconventional word representations, like the one posited here, require less training data in order to generalize successfully. On sets with 5 training strings, the mln based on the conventional model learned the generalizations on 3 out of the 10 sets. On the other hand, the mln based on the unconventional model learned the generalizations on 9 out of the 10 sets. On sets with 10 training strings, the mln based on the conventional model learned the generalizations on 6 out of the 10 sets. The mln based on the unconventional model learned the generalization on all 10 sets.

## 9. Robotic planning

Unconventional word models can potentially reduce the planning complexity of cooperative groups of *heterogeneous* robots (i.e., groups of two or more robots with non-identical functionality). In such a system, robots interact to perform tasks that would be impossible for any single agent to complete in isolation. Attempting to account for *all* different possible interactions, the representative dfa generated by classical automata operations is typically large. In this section, it is demonstrated that unconventional word models may provide compact interaction representations and permit computational savings, both in planning, but primarily in learning these models.

### 9.1. Planning case study

**Figure 5:**
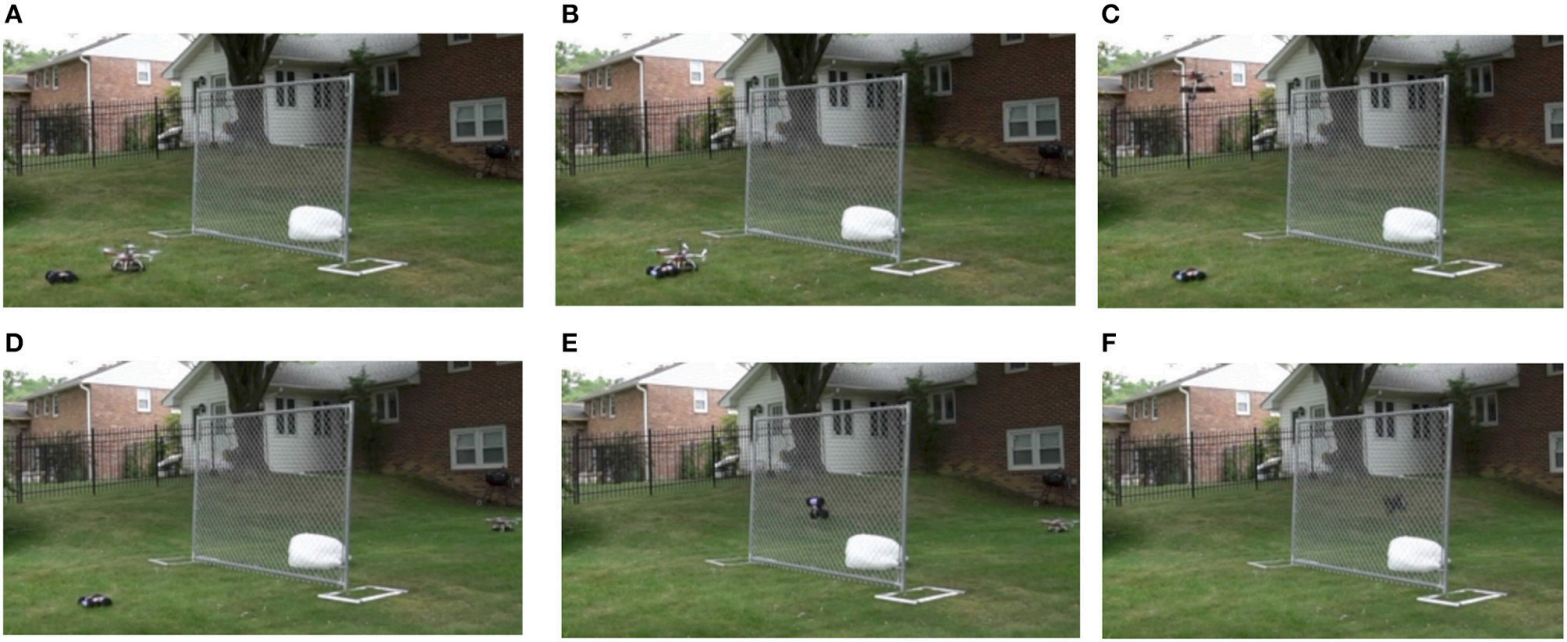
The heterogeneous robotic system considered in this section. The two robots can latch onto each other by means of a powered spool mechanism. By positioning itself on the other side of the fence, the quadrotor can act as an anchor point for the crawler, which will use its powered spool to reel itself up and over the fence to reach the other side. **(A)** the quadrotor lands in front of the ground vehicle; **(B)** the tip of the ground vehicle's spool attaches to the quadrotor's velcro apron; **(C)** the quadrotor takes off to fly over the fence, tethered on the ground vehicle which lets the line real out; **(D)** the quadrotor lands on the other side of the fence; **(E)** the ground robot uses its powered spool to reel in the line and climb vertically against the fence; **(F)** the ground robot has made it over the fence and is on its way to the soft landing area on the other side of the fence.

Consider a heterogeneous robotic system consisting of two vehicles: a ground vehicle (referred to as the *crawler*) and an aerial vehicle (referred to as the *quadrotor*). These two vehicles are able to operate independently, in isolation, or can be connected together by a flexible *tether*. The quadrotor is also able to perch on a flat surface, and use the latter as an anchor point when tethered to the crawler. Once the perching occurs, the crawler is able to reel in the tether, allowing for traversal of obstacles in order to reach a desired location (Figure 5). It is assumed that the crawler cannot traverse certain obstacles in its environment, such as a steep hill, without an applying force to the anchored tether.

The primary motivation in using an unconventional word model is enabling the heterogeneous system to autonomously traverse a variety of otherwise insurmountable obstacles (e.g., the fence in Figure 5) after a small amount of training. The training data in this case would be a human operator manually controlling the quadrotor and crawler to allow the latter to climb over the fence using its spool. Using operator coordinating decisions as data, the system learns which action sets are most likely to result in the crawler reaching its goal position. Another potential benefit of the unconventional model is a reduction in the calculation times which allow for the automated planner to better determine the best course of action in real time.

Table 9 shows the alphabet used for the conventional model, and the corresponding properties that become the unary relations in the unconventional model. Overall, these properties encode three separate pieces of information: the vehicle under concern (crawler/quad), the motion it makes (move/stop), and whether it is tethered, untethered, or attaching. Note that attach only co-occurs with crawler and move.

**Table 9:** Feature geometry for each state of the heterogeneous multi-robot system of Figure 5.

**Conventional Alphabet**	**Vehicle (crawler/quad)**	**Motion (move/stop)**	**Tether untethered/attach/ethered)**
a	crawler	move	untethered
b	crawler	stop	untethered
c	quad	move	untethered
d	quad	stop	untethered
t	crawler	move	attach
A	crawler	move	tethered
B	crawler	stop	tethered
C	quad	move	tethered
D	quad	stop	tethered

**Figure 6:**
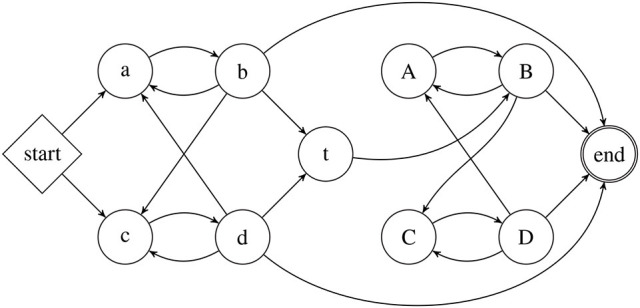
The automaton that accepts strings of robot actions, along cooperative plans in which one robot moves at any given time instant.

The grammar for the cooperative robot behavior is created based on three assumptions: (i) two vehicles cannot move at the same time — one has to stop for the other to start, (ii) the crawler is *tethered* to the quadrotor after attach, and (iii) the strings have to start with move and end with stop. Based on these assumptions, the dfa of Figure 6 is constructed, whose underlying structure is Strictly 2-Local. Transitions in the diagram are unlabeled because, as in Section 7, all transitions are of the form δ(*q*, σ) = σ. Similarly to the strings in Section 7, strings obtained from this dfa are also augmented with initial (⋊) and final (⋉).

To illustrate, the similarity and differences between the conventional and unconventional models, Table 10 shows how the string *abtBCD* would be represented in each of the database files.

**Table 10:** Conventional and unconventional word models consistent with the word (plan) *abtBCD*.

**Conventional word model**	**Unconventional word model**
initial(*A*)	initial(*A*)
a(*B*)	crawler(*B*), move(*B*), untethered(*B*)
b(*C*)	crawler(*C*), move(*C*), untethered(*C*)
t(*D*)	crawler(*D*), move(*D*), attach(*D*)
B(*E*)	crawler(*E*), stop(*E*), tethered(*E*)
C(*F*)	quad(*F*), move(*F*), tethered(*F*)
D(*G*)	quad(*G*), stop(*G*), tethered(*G*)
final(*H*)	final(*H*)
adjacent(*A, B*)	adjacent(*A, B*)
adjacent(*B, C*)	adjacent(*B, C*)
adjacent(*C, D*)	adjacent(*C, D*)
adjacent(*D, E*)	adjacent(*D, E*)
adjacent(*E, F*)	adjacent(*E, F*)
adjacent(*F, G*)	adjacent(*F, G*)
adjacent(*G, H*)	adjacent(*G, H*)

The grammar of the language generated by the dfa of Figure 6 is expressed as a list of forbidden 2-factors in Table 11. In this table, we only listed unique 2-factors for the conventional model, even though one 2-factor in the unconventional one might correspond to several forbidden 2-factors in the conventional model. For example, both untethered-tethered and move-move forbid the substring aA, but it is only listed for untethered-tethered.

**Table 11:** Forbidden 2-factors constituting the strictly 2-Local grammar for the cooperative behavior of the heterogeneous robotic system of Figure 5, under the Conventional and Unconventional Word Models.

**Unconventional model**	**Conventional model**
initial-stop	⋊b, ⋊d, ⋊B, ⋊D
initial-attach	⋊t
initial-tethered	⋊A, ⋊B, ⋊C, ⋊D
move-final	a⋉, c⋉, t⋉, A⋉, C⋉
untethered-tethered	aA, aB, aC, aD, bA, bB, bC, bD, cA, cB, cC, cD, dA, dB, dC, dD
tethered-untethered	Aa, Ab, Ac, Ad, Ba, Bb, Bc, Bd, Ca, Cb, Cc, Cd, Da, Db, Dc, Dd
move-move	aa, cc, ac, at, ca, ct, tt, tA, tC, AA, CC, At, AC, Ct, CA
stop-stop	bb, dd, bd, db, BB, DD BD, DB
attach-untethered	tb, td
tethered-attach	Bt, Dt
[move, crawler][stop, quad]	ad, AD
[move, quad][stop, crawler]	cb, CB

### 9.2. Markov logic networks with conventional and unconventional models

The formulas in the mln with the conventional model included the statements like those presented in Section 6 for Strictly 2-Local grammars, in addition to statements with *initial* and *final* as in Section 7. This resulted in a total of 99 statements. The .mln file, which lists all these statements, is given in the Appendix.

For the unconventional model, the formulas can be categorized into three types as listed below, yielding a total of 39 statements. These statements were selected based on our own knowledge that the tether features do not interact with the vehicle and motion features, but that vehicle and motion features do interact, as only one vehicle was allowed to move at any point. Thus, the statements are (i) 9 fo statements involving the tether features {tethered, untethered, attach}, (ii) 16 fo statements involving combinations of motion and vehicle features, and (iii) 14 fo statements involving individual feature combined with initial and final. The .mln file with the complete list is given in the Appendix.

The runtime of the weight-learning algorithm for the robot grammar is given in Table 12. Learning weights for the unconventional model took about half the time compared to doing the same for the conventional model.

**Table 12:** Runtime for learning weights for robotic statements.

	**Conventional model**	**Unconventional model**
20 strings	1 min, 3.89s	35.7s
50 strings	8 min, 57.5s	4 min, 16.3s
100 strings	3 h, 3.07 min	28 min, 49.6s
250 strings	11 h, 46.8 min	5 h, 6.57 min

### 9.3. Training data

We generated strings for the training data-set, assuming that all transitions have the same probability. We considered training data sets of 5, 10, 20, 50, 100, and 250 strings. For each of the data sets of size 5 and 10, we generated 10 files. Due to the significantly longer list of statements that Alchemy had to assign weights to (and consequently longer runtime for training), we did not train it on multiple files of sizes 20, 50, 100, and 250.

### 9.4. Evaluation method and results

The learning outcomes under the conventional and unconventional mln models on 20 training strings are presented in Tables 13, 14, respectively. For instance, the entry in row *a* and column *b* expresses the conditional probability P(b(*x*)|a(*x*), adjacent(*x, y*)), which corresponds to ρ(*a, b*) in the associated pdfa of Figure 6. We introduced a threshold of 0.05 for the probability of allowed bi-grams. The allowed bi-grams (based on the threshold) are shaded in the table.

**Table 13:** Conventional model trained on 20 training strings.

	**a**	**b**	**c**	**d**	**t**	**A**	**B**	**C**	**D**	**⋉**
⋊	0.496927	0.000815	0.493377	0.000592	0.001016	0.001278	0.000736	0.001084	0.001039	0.003137
a	6.54E-05	0.999517	5.08E-05	2.17E-06	0.000101	0.000104	7.17E-06	8.87E-05	3.41E-05	2.96E-05
b	0.094095	0.008997	0.091477	0.007491	0.367243	0.000349	0.007735	0.010931	0.009811	0.401872
c	8.23E-05	4.03E-06	6.22E-05	0.999358	0.000145	0.000147	7.99E-06	0.000119	4.02E-05	3.35E-05
d	0.071082	0.006026	0.085054	0.004911	0.522097	0.000112	0.005017	0.00774	0.006597	0.291364
t	0.000247	4.43E-05	0.00021	3.24E-05	0.000324	0.000334	0.998374	0.00029	4.29E-05	0.000101
A	0.000329	9.12E-05	0.000283	7.63E-05	0.00041	0.000423	0.997791	0.000381	5.96E-05	0.000156
B	0.00242	0.00283	0.001839	0.002265	8.07E-05	0.15365	0.002296	0.054035	0.00315	0.777433
C	8.88E-05	4.82E-05	7.07E-05	3.93E-05	0.000125	0.000131	9.83E-06	0.000113	0.999327	4.67E-05
D	0.008202	0.009073	0.006643	0.00762	0.000387	0.286747	0.00799	0.245995	0.009906	0.417436

**Table 14:** Unconventional model trained on 20 training strings.

	**a**	**b**	**c**	**d**	**t**	**A**	**B**	**C**	**D**	**⋊**
⋉	0.503601	0.000828	0.494177	0.000812	0.000217	0.000181	2.97E-07	0.000178	2.92E-07	4.84E-06
a	1.32E-04	0.99942	2.64E-04	2.35E-06	0.000107	4.22E-09	3.20E-05	8.45E-09	7.52E-11	4.30E-05
b	0.29998	0.00156	0.244344	0.003385	0.242705	9.60E-06	4.99E-08	7.82E-06	1.08E-07	0.208008
c	2.65E-04	2.57E-06	1.34E-04	0.999328	0.000214	8.48E-09	8.22E-11	4.28E-09	3.20E-05	2.46E-05
d	0.321375	0.002458	0.324781	0.001342	0.260015	1.03E-05	7.87E-08	1.04E-05	4.30E-08	0.090007
t	3.60E-09	2.73E-05	7.20E-09	6.41E-11	7.34E-09	0.000132	0.999575	0.000264	2.35E-06	1.19E-08
A	2.00E-08	1.52E-04	4.01E-08	3.57E-10	6.31E-09	0.000132	0.999328	0.000264	2.35E-06	0.000122
B	4.01E-05	2.09E-07	3.27E-05	4.53E-07	1.26E-05	0.263762	0.001372	0.214843	0.002976	0.516961
C	4.03E-08	3.90E-10	2.03E-08	1.52E-04	1.27E-08	0.000265	2.57E-06	0.000134	0.999377	6.96E-05
D	5.40E-05	4.13E-07	5.46E-05	2.26E-07	1.70E-05	0.355314	0.002718	0.359079	0.001484	0.281278

These results indicate that both models meet this benchmark of success with 20 training strings. Generally, however, the unconventional model provides higher probabilities to licit sequences.

To evaluate how much training is needed for each model to reliably generalize, we tested both models on small training samples. Specifically, we tested both models on ten training sets with 10 strings and ten training sets with 5 strings. On sets with 5 training strings, the trained mln with the conventional model learns the correct grammar on 1 set out of 10. The trained mln with the unconventional model learns the correct grammar list on 6 sets out of 10. On sets with 10 training strings, conventional models learn the correct grammar only on 4 sets out of 10. The unconventional model learns the correct grammar on 9 sets out of 10.

The empirical conclusions from this case study are in agreement with those of Section 8: mlns trained with unconventional models seem to require less training data to converge. Additionally, the computation time required by the unconventional model is far smaller than that of the conventional one, since the former featured a significantly more compact representation.

## 10. Discussion

This article has applied statistical relational learning to the problem of inferring categorical and stochastic formal languages, a problem typically identified with the field of grammatical inference. The rationale for tackling these learning problems with relational learning is that the learning techniques separate issues of representation from issues of inference. In this way, domain-specific knowledge can be incorporated into the representations of strings when appropriate.

Our case studies indicate that not only can mlns mimic traditional n-gram language models, but that successful inference with unconventional word models, which permit multiple positions in strings to share properties, concretely improve inference. This is because with the richer representations unconventional models provide, fewer formulas in the mln are necessary to instantiate a sufficiently expressive parametric model as compared to the representations provided by conventional models. Two important consequences of this are a reduction in the training time and a reduction in the amount of data required to generalize successfully. These results were demonstrated in different domains, phonology and robotics.

In addition to exploring learning with unconventional models in these domains and others, there are four other important avenues for future research.

While this article considered the learning problem of finding weights given formula, another problem is identifying both the formulas and the weights. In this regard, it would be interesting to compare the learning of stochastic formal languages with statistical relational learning methods where the formulas are not provided a priori to their learning with grammatical inference methods such as ALEGRIA (de la Higuera, 2010).

Second, is the problem of scalability. Unless the input files to Alchemy 2 were small, this software required large computational resources in terms of time and memory. Developing better software and algorithms to allow mlns to scale is essential to moving from the examples presented here to more complex real-world applications. We suspect that mlns instantiated by formulas of the type discussed in this paper can be brought to scale. This is because the notion of sub-structure which underlies these methods provides a generality relation which structures the hypothesis space and thus significantly cuts down the computational resources required (De Raedt, 2008). Studying how to integrate this inference structure into mlns with the right properties would be a worthwhile endeavor.

Third, Section 7.3 introduces a way to translate the weights on the formulas in the mln to probabilities on the transitions in a pdfa. A welcome theoretical result would be to establish the general conditions and algorithmic procedure under which this translation can occur and be computed automatically.

Finally, while the case studies in this article are experimental, we believe that general theoretical results relating relational learning, grammatical inference, unconventional word models, and formal languages are now within reach. We hope that the present paper spurs such research activity.

## Author contributions

MV developed the training data and the mlns used in sections 7–9. AZ conducted the analysis of the results in sections 7–9. Everyone contributed equally to the design of the experiments in sections 7–9. JH, HT, and KS-G drafted section 1; JH sections 2, 4; HT and JH sections 3 and 5; MV section 6; AZ, MV, and JH section 7; KS-G, MV, AZ, and JH section 8; HT, MS, MV, AZ and JH section 9; and JH section 10. Everyone helped revise the initial draft.

### Conflict of interest statement

The authors declare that the research was conducted in the absence of any commercial or financial relationships that could be construed as a potential conflict of interest.

## Footnotes

^1^Model signatures may also include constants, but we leave out this term for two reasons. First, within model theory, constants are often treated as functions which take zero arguments. Second, the term constant has a different meaning in the statistical relational learning literature. There, constants are understood as domain elements which ground formulas.

^2^Syllable weights, and in fact stress itself, manifest differently in different languages. We abstract away from this fact.

^3^The latter stringset equals Σ^*^ĹΣ^*^∪Σ^*^$\overset{\prime }{\text{H}}$Σ^*^.

## Appendix:

### Input .mln files for Alchemy 2

#### ABC-strings

a(char)
b(char)
c(char)
initial(char)
final(char)
adjacent(char,char)
 
 
0 adjacent(x,y) ^ initial(x) ^ a(y)
0 adjacent(x,y) ^ initial(x) ^ b(y)
0 adjacent(x,y) ^ initial(x) ^ c(y)
0 adjacent(x,y) ^ initial(x) ^ final(y)
0 adjacent(x,y) ^ a(x) ^ final(y)
0 adjacent(x,y) ^ b(x) ^ final(y)
0 adjacent(x,y) ^ c(x) ^ final(y)
0 adjacent(x,y) ^ a(x) ^ a(y)
0 adjacent(x,y) ^ a(x) ^ b(y)
0 adjacent(x,y) ^ a(x) ^ c(y)
0 adjacent(x,y) ^ b(x) ^ a(y)
0 adjacent(x,y) ^ b(x) ^ b(y)
0 adjacent(x,y) ^ b(x) ^ c(y)
0 adjacent(x,y) ^ c(x) ^ a(y)
0 adjacent(x,y) ^ c(x) ^ b(y)
0 adjacent(x,y) ^ c(x) ^ c(y)
 
 

#### Unbounded stress pattern

##### Conventional model

h(char)
l(char)
hstr(char)
lstr(char)
follows(char,char)
hasLstr(string)
hasHstr(string)
isString(string)
 
 
0 follows(x,y)^ h(x) ^ h(y)
0 follows(x,y)^ h(x) ^ l(y)
0 follows(x,y)^ h(x) ^ hstr(y)
0 follows(x,y)^ h(x) ^ lstr(y)
0 follows(x,y)^ l(x) ^ h(y)
0 follows(x,y)^ l(x) ^ l(y)
0 follows(x,y)^ l(x) ^ hstr(y)
0 follows(x,y)^ l(x) ^ lstr(y)
0 follows(x,y)^ hstr(x) ^ h(y)
0 follows(x,y)^ hstr(x) ^ l(y)
0 follows(x,y)^ hstr(x) ^ hstr(y)
0 follows(x,y)^ hstr(x) ^ lstr(y)
0 follows(x,y)^ lstr(x) ^ h(y)
0 follows(x,y)^ lstr(x) ^ l(y)
0 follows(x,y)^ lstr(x) ^ hstr(y)
0 follows(x,y)^ lstr(x) ^ lstr(y)
0 isString(x) ^ (hasLstr(x) v hasHstr(x))
 
 

##### Unconventional model

follows(char,char)
h(char)
str(char)
l(char)
hasStress(string)
isString(string)
 
 
0 follows(x,y) ^ h(x) ^ str(x) ^ h(y)
0 follows(x,y) ^ h(x) ^ str(x) ^ l(y)
0 follows(x,y) ^ h(x) ^ str(x) ^ str(y)
0 follows(x,y) ^ l(x) ^ str(x) ^ h(y)
0 follows(x,y) ^ l(x) ^ str(x) ^ l(y)
0 follows(x,y) ^ l(x) ^ str(x) ^ str(y)
0 follows(x,y) ^ h(x) ^ h(y)
0 follows(x,y) ^ h(x) ^ l(y)
0 follows(x,y) ^ h(x) ^ str(y)
0 follows(x,y) ^ l(x) ^ h(y)
0 follows(x,y) ^ l(x) ^ l(y)
0 follows(x,y) ^ l(x) ^ str(y)
0 follows(x,y) ^ str(x) ^ h(y)
0 follows(x,y) ^ str(x) ^ l(y)
0 follows(x,y) ^ str(x) ^ str(y)
0 isString(x) ^ hasStress(x)
 
 

#### Robot planning

##### Conventional model

adjacent(char,char)
initial(char)
final(char)
a(char)
b(char)
c(char)
d(char)
a-prime(char)
b-prime(char)
c-prime(char)
d-prime(char)
t(char)
 
 
0 adjacent(x,y) ^ a(x) ^ a(y)
0 adjacent(x,y) ^ a(x) ^ b(y)
0 adjacent(x,y) ^ a(x) ^ c(y)
0 adjacent(x,y) ^ a(x) ^ d(y)
0 adjacent(x,y) ^ a(x) ^ a-prime(y)
0 adjacent(x,y) ^ a(x) ^ b-prime(y)
0 adjacent(x,y) ^ a(x) ^ c-prime(y)
0 adjacent(x,y) ^ a(x) ^ d-prime(y)
0 adjacent(x,y) ^ a(x) ^ t(y)
0 adjacent(x,y) ^ b(x) ^ b(y)
0 adjacent(x,y) ^ b(x) ^ a(y)
0 adjacent(x,y) ^ b(x) ^ c(y)
0 adjacent(x,y) ^ b(x) ^ d(y)
0 adjacent(x,y) ^ b(x) ^ a-prime(y)
0 adjacent(x,y) ^ b(x) ^ b-prime(y)
0 adjacent(x,y) ^ b(x) ^ c-prime(y)
0 adjacent(x,y) ^ b(x) ^ d-prime(y)
0 adjacent(x,y) ^ b(x) ^ t(y)
0 adjacent(x,y) ^ c(x) ^ c(y)
0 adjacent(x,y) ^ c(x) ^ a(y)
0 adjacent(x,y) ^ c(x) ^ b(y)
0 adjacent(x,y) ^ c(x) ^ d(y)
0 adjacent(x,y) ^ c(x) ^ a-prime(y)
0 adjacent(x,y) ^ c(x) ^ b-prime(y)
0 adjacent(x,y) ^ c(x) ^ c-prime(y)
0 adjacent(x,y) ^ c(x) ^ d-prime(y)
0 adjacent(x,y) ^ c(x) ^ t(y)
0 adjacent(x,y) ^ d(x) ^ d(y)
0 adjacent(x,y) ^ d(x) ^ a(y)
0 adjacent(x,y) ^ d(x) ^ b(y)
0 adjacent(x,y) ^ d(x) ^ c(y)
0 adjacent(x,y) ^ d(x) ^ a-prime(y)
0 adjacent(x,y) ^ d(x) ^ b-prime(y)
0 adjacent(x,y) ^ d(x) ^ c-prime(y)
0 adjacent(x,y) ^ d(x) ^ d-prime(y)
0 adjacent(x,y) ^ d(x) ^ t(y)
0 adjacent(x,y) ^ a-prime(x) ^ a-prime(y)
0 adjacent(x,y) ^ a-prime(x) ^ a(y)
0 adjacent(x,y) ^ a-prime(x) ^ b(y)
0 adjacent(x,y) ^ a-prime(x) ^ c(y)
0 adjacent(x,y) ^ a-prime(x) ^ d(y)
0 adjacent(x,y) ^ a-prime(x) ^ b-prime(y)
0 adjacent(x,y) ^ a-prime(x) ^ c-prime(y)
0 adjacent(x,y) ^ a-prime(x) ^ d-prime(y)
0 adjacent(x,y) ^ a-prime(x) ^ t(y)
0 adjacent(x,y) ^ b-prime(x) ^ b-prime(y)
0 adjacent(x,y) ^ b-prime(x) ^ a(y)
0 adjacent(x,y) ^ b-prime(x) ^ b(y)
0 adjacent(x,y) ^ b-prime(x) ^ c(y)
0 adjacent(x,y) ^ b-prime(x) ^ d(y)
0 adjacent(x,y) ^ b-prime(x) ^ a-prime(y)
0 adjacent(x,y) ^ b-prime(x) ^ c-prime(y)
0 adjacent(x,y) ^ b-prime(x) ^ d-prime(y)
0 adjacent(x,y) ^ b-prime(x) ^ t(y)
0 adjacent(x,y) ^ c-prime(x) ^ c-prime(y)
0 adjacent(x,y) ^ c-prime(x) ^ a(y)
0 adjacent(x,y) ^ c-prime(x) ^ b(y)
0 adjacent(x,y) ^ c-prime(x) ^ c(y)
0 adjacent(x,y) ^ c-prime(x) ^ d(y)
0 adjacent(x,y) ^ c-prime(x) ^ a-prime(y)
0 adjacent(x,y) ^ c-prime(x) ^ b-prime(y)
0 adjacent(x,y) ^ c-prime(x) ^ d-prime(y)
0 adjacent(x,y) ^ c-prime(x) ^ t(y)
0 adjacent(x,y) ^ d-prime(x) ^ d-prime(y)
0 adjacent(x,y) ^ d-prime(x) ^ a(y)
0 adjacent(x,y) ^ d-prime(x) ^ b(y)
0 adjacent(x,y) ^ d-prime(x) ^ c(y)
0 adjacent(x,y) ^ d-prime(x) ^ d(y)
0 adjacent(x,y) ^ d-prime(x) ^ a-prime(y)
0 adjacent(x,y) ^ d-prime(x) ^ b-prime(y)
0 adjacent(x,y) ^ d-prime(x) ^ c-prime(y)
0 adjacent(x,y) ^ d-prime(x) ^ t(y)
0 adjacent(x,y) ^ t(x) ^ t(y)
0 adjacent(x,y) ^ t(x) ^ a(y)
0 adjacent(x,y) ^ t(x) ^ b(y)
0 adjacent(x,y) ^ t(x) ^ c(y)
0 adjacent(x,y) ^ t(x) ^ d(y)
0 adjacent(x,y) ^ t(x) ^ a-prime(y)
0 adjacent(x,y) ^ t(x) ^ b-prime(y)
0 adjacent(x,y) ^ t(x) ^ c-prime(y)
0 adjacent(x,y) ^ t(x) ^ d-prime(y)
0 adjacent(x,y) ^ initial(x) ^ a(y)
0 adjacent(x,y) ^ initial(x) ^ b(y)
0 adjacent(x,y) ^ initial(x) ^ c(y)
0 adjacent(x,y) ^ initial(x) ^ d(y)
0 adjacent(x,y) ^ initial(x) ^ a-prime(y)
0 adjacent(x,y) ^ initial(x) ^ b-prime(y)
0 adjacent(x,y) ^ initial(x) ^ c-prime(y)
0 adjacent(x,y) ^ initial(x) ^ d-prime(y)
0 adjacent(x,y) ^ initial(x) ^ t(y)
0 adjacent(x,y) ^ a(x) ^ final(y)
0 adjacent(x,y) ^ b(x) ^ final(y)
0 adjacent(x,y) ^ c(x) ^ final(y)
0 adjacent(x,y) ^ d(x) ^ final(y)
0 adjacent(x,y) ^ a-prime(x) ^ final(y)
0 adjacent(x,y) ^ b-prime(x) ^ final(y)
0 adjacent(x,y) ^ c-prime(x) ^ final(y)
0 adjacent(x,y) ^ d-prime(x) ^ final(y)
0 adjacent(x,y) ^ t(x) ^ final(y)
 
 

##### Unconventional model

adjacent(char,char)
crawler(char)
quad(char)
move(char)
stop(char)
tethered(char)
untethered(char)
attach(char)
initial(char)
final(char)
 
 
 
 
 
 
0 adjacent(x,y) ^ crawler(x) ^ move(x) ^ crawler(y) ^ move(y)
0 adjacent(x,y) ^ crawler(x) ^ move(x) ^ crawler(y) ^ stop(y)
0 adjacent(x,y) ^ crawler(x) ^ move(x) ^ quad(y) ^ move(y)
0 adjacent(x,y) ^ crawler(x) ^ move(x) ^ quad(y) ^ stop(y)
0 adjacent(x,y) ^ crawler(x) ^ stop(x) ^ crawler(y) ^ move(y)
0 adjacent(x,y) ^ crawler(x) ^ stop(x) ^ crawler(y) ^ stop(y)
0 adjacent(x,y) ^ crawler(x) ^ stop(x) ^ quad(y) ^ move(y)
0 adjacent(x,y) ^ crawler(x) ^ stop(x) ^ quad(y) ^ stop(y)
0 adjacent(x,y) ^ quad(x) ^ move(x) ^ crawler(y) ^ move(y)
0 adjacent(x,y) ^ quad(x) ^ move(x) ^ crawler(y) ^ stop(y)
0 adjacent(x,y) ^ quad(x) ^ move(x) ^ quad(y) ^ move(y)
0 adjacent(x,y) ^ quad(x) ^ move(x) ^ quad(y) ^ stop(y)
0 adjacent(x,y) ^ quad(x) ^ stop(x) ^ crawler(y) ^ move(y)
0 adjacent(x,y) ^ quad(x) ^ stop(x) ^ crawler(y) ^ stop(y)
0 adjacent(x,y) ^ quad(x) ^ stop(x) ^ quad(y) ^ move(y)
0 adjacent(x,y) ^ quad(x) ^ stop(x) ^ quad(y) ^ stop(y)
0 adjacent(x,y) ^ untethered(x) ^ untethered(y)
0 adjacent(x,y) ^ untethered(x) ^ tethered(y)
0 adjacent(x,y) ^ tethered(x) ^ untethered(y)
0 adjacent(x,y) ^ tethered(x) ^ tethered(y)
0 adjacent(x,y) ^ attach(x) ^ untethered(y)
0 adjacent(x,y) ^ untethered(x) ^ attach(y)
0 adjacent(x,y) ^ attach(x) ^ tethered(y)
0 adjacent(x,y) ^ tethered(x) ^ attach(y)
0 adjacent(x,y) ^ initial(x) ^ crawler(y)
0 adjacent(x,y) ^ initial(x) ^ quad(y)
0 adjacent(x,y) ^ initial(x) ^ move(y)
0 adjacent(x,y) ^ initial(x) ^ stop(y)
0 adjacent(x,y) ^ initial(x) ^ untethered(y)
0 adjacent(x,y) ^ initial(x) ^ tethered(y)
0 adjacent(x,y) ^ initial(x) ^ attach(y)
0 adjacent(x,y) ^ crawler(x) ^ final(y)
0 adjacent(x,y) ^ quad(x) ^ final(y)
0 adjacent(x,y) ^ move(x) ^ final(y)
0 adjacent(x,y) ^ stop(x) ^ final(y)
0 adjacent(x,y) ^ tethered(x) ^ final(y)
0 adjacent(x,y) ^ untethered(x) ^ final(y)
0 adjacent(x,y) ^ attach(x) ^ final(y)
 
 
